# Rapid Anti-Inflammatory Effects of Gonadotropin-Releasing Hormone Antagonism in Rheumatoid Arthritis Patients with High Gonadotropin Levels in the AGRA Trial

**DOI:** 10.1371/journal.pone.0139439

**Published:** 2015-10-13

**Authors:** Anita Kåss, Ivana Hollan, Morten Wang Fagerland, Hans Christian Gulseth, Peter Abusdal Torjesen, Øystein Torleiv Førre

**Affiliations:** 1 Institute of Clinical Medicine, Faculty of Medicine, University of Oslo, Oslo, Norway; 2 Department of Rheumatology, Betanien Hospital, Skien, Norway; 3 Department of Rheumatology, Lillehammer Hospital for Rheumatic Diseases, Lillehammer, Norway; 4 Unit of Biostatistics and Epidemiology, Oslo University Hospital, Oslo, Norway; 5 Hormone Laboratory, Oslo University Hospital, Oslo, Norway; 6 Department of Rheumatology, Oslo University Hospital, Oslo, Norway; VU University Medical Center, NETHERLANDS

## Abstract

**Objectives:**

Gonadotropin-releasing hormone (GnRH) and pituitary gonadotropins, which appear to be proinflammatory, undergo profound secretory changes during events associated with rheumatoid arthritis (RA) onset, flares, or improvement e.g. menopausal transition, postpartum, or pregnancy. Potential anti-inflammatory effects of GnRH-antagonists may be most pronounced in patients with high GnRH and gonadotropin levels. Therefore, we investigated the efficacy and safety of a GnRH-antagonist, cetrorelix, in RA patients with high gonadotropin levels.

**Methods:**

We report intention-to-treat post hoc analyses among patients with high gonadotropin levels (N = 53), i.e. gonadotropin levels>median, from our proof-of-concept, double-blind AGRA-study (N = 99). Patients with active longstanding RA, randomized to subcutaneous cetrorelix (5mg days1–2; 3mg days 3–5) or placebo, were followed through day 15. Only predefined primary and secondary endpoints were analyzed.

**Results:**

The primary endpoint, Disease Activity Score of 28-joint counts with C-reactive protein (DAS28-CRP), improved with cetrorelix compared with placebo by day 5 (-1.0 vs. -0.4, P = 0∙010). By day 5, more patients on cetrorelix achieved at least a 20% improvement in the American College of Rheumatology scale (44% vs. 19%, P = 0.049), DAS28-CRP≤3.2 (24% vs. 0%, P = 0.012), and European League against Rheumatism ‘Good-responses’ (19% vs. 0%, P = 0.026). Tumor necrosis factor-α, interleukin-1β, interleukin-10, and CRP decreased with cetrorelix (P = 0.045, P = 0.034, P = 0.020 and P = 0.042 respectively) compared with placebo by day 15. Adverse event rates were similar between groups.

**Conclusions:**

GnRH-antagonism produced rapid anti-inflammatory effects in RA patients with high gonadotropin levels. GnRH should be investigated further in RA.

**Trial Registration:**

ClinicalTrials.gov NCT00667758

## Introduction

Hypothalamic and pituitary hormones are key regulators of the entire endocrine system. The hypothalamic-pituitary-gonadal axis refers to the pulsatile hypothalamic gonadotropin-releasing hormone (GnRH), which stimulates the secretion of the pituitary gonadotropins, luteinizing hormone (LH) and follicle-stimulating hormone (FSH), which, in turn, stimulate the production of gonadal hormones, such as oestrogen and testosterone.

Rheumatoid arthritis (RA) symptoms may develop or flare during stimulation of the hypothalamic-pituitary-gonadal axis, when GnRH and gonadotropin secretion increases, such as during the menopausal transition, postpartum, anti-oestrogen therapy, or polycystic ovarian syndrome (PCOS) [1–4]. In contrast, improvement in RA disease activity is associated with suppression of the hypothalamic-pituitary-gonadal axis, such as in pregnancy or fasting [5,6].

Due to these observations, there has been extensive research investigating potential therapeutic effects of the gonadal hormones, oestrogen and testosterone; showing, at best, a modest effect on RA disease activity. However, hypothalamic GnRH and pituitary gonadotropins, which regulate gonadal hormones, have hardly been studied in RA.

In-vitro and animal studies in both sexes indicate that GnRH and gonadotropins are proinflammatory. Remarkably, human T cells can secrete immunoactive GnRH [7], which directly activates T cells through GnRH receptors in an autocrine and paracrine, cytokine-like, clinically meaningful way [8–12]. GnRH may also act indirectly on immune cells through LH [13] and/or FSH [14]. Indeed, GnRH-agonists have been associated with the onset of RA [15]. Thus, inhibiting GnRH, consequently inhibiting gonadotropins, might have therapeutic potential in RA. Currently, GnRH-antagonists are primarily used for the reduction of gonadotropins and gonadal hormones, as in infertility treatment and prostate cancer.

The Antagonist to Gonadotropin-Releasing Hormone in Rheumatoid Arthritis (AGRA) study investigated the safety and efficacy of a GnRH-antagonist, cetrorelix (Cetrotide, Merck Serono), in RA [16]. GnRH-antagonists essentially suppress the hypothalamic-pituitary-gonadal axes in both sexes, and had not been tested in human autoimmune disease before. Therefore, for ethical reasons, the AGRA study was short. Moreover, we hypothesized that if rapid hormonal changes could lead to RA flares as in the postpartum period, then opposing rapid hormonal changes could lead to rapid amelioration by hormone-targeting therapy. In the AGRA study, cetrorelix treatment led to improvements of RA disease activity and a reduction of tumor necrosis factor-α (TNF-α) level, already by day 5. In that study, we also found that TNF-α levels were more strongly associated with gonadotropin levels than oestradiol levels. TNF-α levels were not associated with testosterone or cortisol levels. Therefore we hypothesized that GnRH antagonism would be particularly effective at reducing key cytokines and disease activity in patients with high gonadotropins. This was considered to be biologically plausible because of the close association between TNF-α and gonadotropins in RA patients, and the fact that GnRH antagonism primarily reduces gonadotropins.

High gonadotropin levels are observed in postmenopausal women, who represent about 60–70% of RA patients [17]. Women are more prone to RA, generally have more severe disease, and they have significantly poorer responses to conventional medications than men [18]. These gender differences may be partially explained by hormonal factors.

Taken together, novel treatment options targeting hormones should be carefully investigated, and may also simultaneously increase our understanding of the pathophysiology of RA. Due to these facts, we considered it important to perform post hoc analyses of the AGRA study to investigate the efficacy and safety of GnRH-antagonism in RA patients with high gonadotropin levels.

## Methods

The protocol for this trial and supporting CONSORT checklist are available as supporting information; see S1 Protocol and S1 Consort Checklist.

AGRA is a proof-of-concept, investigator-initiated, randomized, double-blind, placebo-controlled trial assessing the effects of GnRH antagonism in RA patients [16]; carried out at Betanien Hospital, Norway; ClinicalTrials.gov number NCT00667758. Herein we report post hoc analyses.

All patients gave written informed consent to participate. The Regional Committee for Medical and Health Research Ethics (South-East Region) approved the study protocol. The study was carried out in compliance with the principles expressed in the Declaration of Helsinki. An independent data-monitoring committee monitored the trial for scientific integrity. The first author wrote all manuscript drafts and all the authors revised the manuscript for intellectual content. All the authors made the decision to submit the manuscript for publication, and vouch for the completeness and accuracy of the data and analyses, and for the fidelity of the study to the protocol.

### Patients

Patients’ visits were between August 1, 2008 and June 11, 2011. Key inclusion criteria for AGRA were males or females aged ≥18 years with RA that had been diagnosed on the basis of the American College of Rheumatology (ACR) 1987 revised criteria [19]; with active, and moderate or severe disease defined as a Disease Activity Score for 28-joint counts based on C-reactive protein [20] (DAS28-CRP) >3.2 and at least two of the following criteria: ≥ 6 painful joints, ≥ 3 swollen joints, ESR ≥ 20mm/h, and a C-reactive protein ≥ 10mg/L). Prednisolone ≤ 7.5mg/day was permitted if stable for at least 4 weeks prior to baseline; NSAIDs were permitted if stable for at least 2 weeks prior to baseline; and disease-modifying anti-rheumatic drugs (DMARDs) were permitted if stable for at least 8 weeks prior to baseline. Tumour necrosis factor-α (TNF- α) inhibitor or other biological agents were not permitted during the trial or within 4 weeks prior to inclusion. Infliximab/adalimumab was not permitted at least 3 months prior to inclusion; Rituximab was not permitted at least 6 months prior to inclusion. Intramuscular, intra-articular or intravenous corticosteroids; any hormone replacement therapy; or oral contraception was not permitted during the trial or within 4 weeks prior to inclusion (full criteria available as supporting information; see S1 Appendix).

During the first three months, patients with disease duration> 36 months or patients taking concomitant NSAIDs and prednisolone were also excluded. Due to slow recruitment, these stricter criteria were removed after the first 6 patients were enrolled. This change was not expected to bias our results, and allowed data generated by this trial to be generalized to a wider RA population.

In these post hoc analyses, we examined a subgroup of AGRA patients with high gonadotropin levels. There is no established cut-off for high LH and/or FSH. Therefore, we defined levels above the median as high (i.e. LH>17.3 IU/L and FSH>34.6 IU/L).

### Design

Visits were between 07:30–09:30 in the morning on days 1 (baseline), 2, 3, 4, 5, 10, and 15. On day 5, an additional visit (visit 5b) occurred between 19:30–21:30 in the evening when the greatest suppression of GnRH and gonadotropins was anticipated. Once a patient had been through the screening process and gave informed consent, the unmasked research nurse obtained randomisation information by calling an offsite central office with no clinical involvement in the trial (Clinical Research Centre, University of Oslo) which randomised the patient through computer generated allocation. Randomisation was in random block sizes and stratified for sex, assigning patients in a 1:1 ratio to receive either daily subcutaneous injections of cetrorelix acetate (5mg/d for the first two days and 3mg/d for the following 3 days, in total 5 consecutive days of medication) or corresponding volumes of saline placebo. These cetrorelix doses were chosen to achieve rapid reductions in GnRH, LH, a surrogate marker for GnRH, and FSH.

This unmasked research nurse was responsible for the preparation of cetrorelix and placebo injections at the nurses’ station away from the patient’s treatment room. Once the study drug was prepared in the syringe, the cetrorelix injection (a colourless odourless liquid) was indistinguishable from the placebo injection. The unmasked research nurse did not do any trial assessments. The research nurses successfully maintained masking of all other study personnel (including healthcare providers and outcome assessors) and participants. The research nurse administered the study drug and kept a written locked record of what drug was given. This record was not shared until the database lock had occurred after the last patient’s final visit.

Any concomitant therapy had to be stable prior to and during the study, and taken at the same time of day. Blinded laboratory assessments were performed in batches after the study’s completion.

### Outcomes

Only our protocol-specified primary and secondary intention-to-treat endpoints of the original AGRA trial were analyzed in these post hoc analyses. The primary endpoint was the baseline-adjusted between-group difference in DAS28-CRP [20] by day 5b. Secondary endpoints included the baseline-adjusted between-group difference in cytokines, proportions of patients achieving at least a 20%, 50%, and 70% improvement on the ACR scale [21], categorical DAS28-CRP (European League Against Rheumatoid Arthritis [EULAR]) responses[20,22], DAS28-CRP≤ 3.2 [20], DAS28-CRP< 2.6 [20], and the incidence of adverse events. Protocol-specified DAS28 outcomes were preferentially CRP-based rather than erythrocyte sedimentation rate (ESR)-based, as CRP usually changes before ESR. Based on the results from our case-control study [23], we assessed TNF-α, interleukin-1β, interleukin-2 and interleukin-10. Multiplex technology (Luminex Inc., Austin, Texas) measured these serum cytokines using high-sensitivity assays (sensitivities <0.5pg/mL, allowing the detection of very low concentrations of cytokines) and identical lots of critical reagent of negligible cross-reactivity <2%, according to manufacturers’ instructions (Bio-Rad, Hercules, California). The intra-assay coefficient of variation (CV) was <10% and the inter-assay CV was <15%. No significant variation was noted between duplicates for any sample. To enable intention-to-treat analyses with all patients N = 53, non-detectable values were set at the lower limit of detection for cytokines, as is common for CRP values. Values were above the lower limit of detection in 22 of 53 patients for tumor necrosis factor-α, 11 of 53 patients for interleukin-1β, 14 of 53 patients for interleukin-2, and 26 of 53 patients for interukin-10.

### Statistical Analysis

The original power calculation is previously reported [16]. Baseline-adjusted between-group differences for all continuous endpoints were assessed with the use of analysis-of-covariance using outcome measurements at predefined time points as response variables, and treatment and baseline measurements as covariates. Dichotomous endpoints were compared with the Pearson chi-square test or Suissa-Shuster exact unconditional test, depending on expected-value distribution [24]. No adjustments for multiple analyses were made, owing to highly correlated endpoints. Due to skewed distributions, we compared between-group changes of natural log-transformed cytokine levels from baseline.

The intention-to-treat population was predefined as all randomised patients who received any study-drug injections. Missing values were <1% and could, as predefined, be imputed with the last-observation-carried-forward. The assumptions of normality needed for analyses were approximately valid. All statistical tests were two-sided (α = 0∙05) and performed with Stata 12 and StatXact 9. Analyses were performed by an offsite statistician who received locked databases from blinded investigators and laboratories, and the allocation key from the offsite central office.

## Results

**Fig 1:**
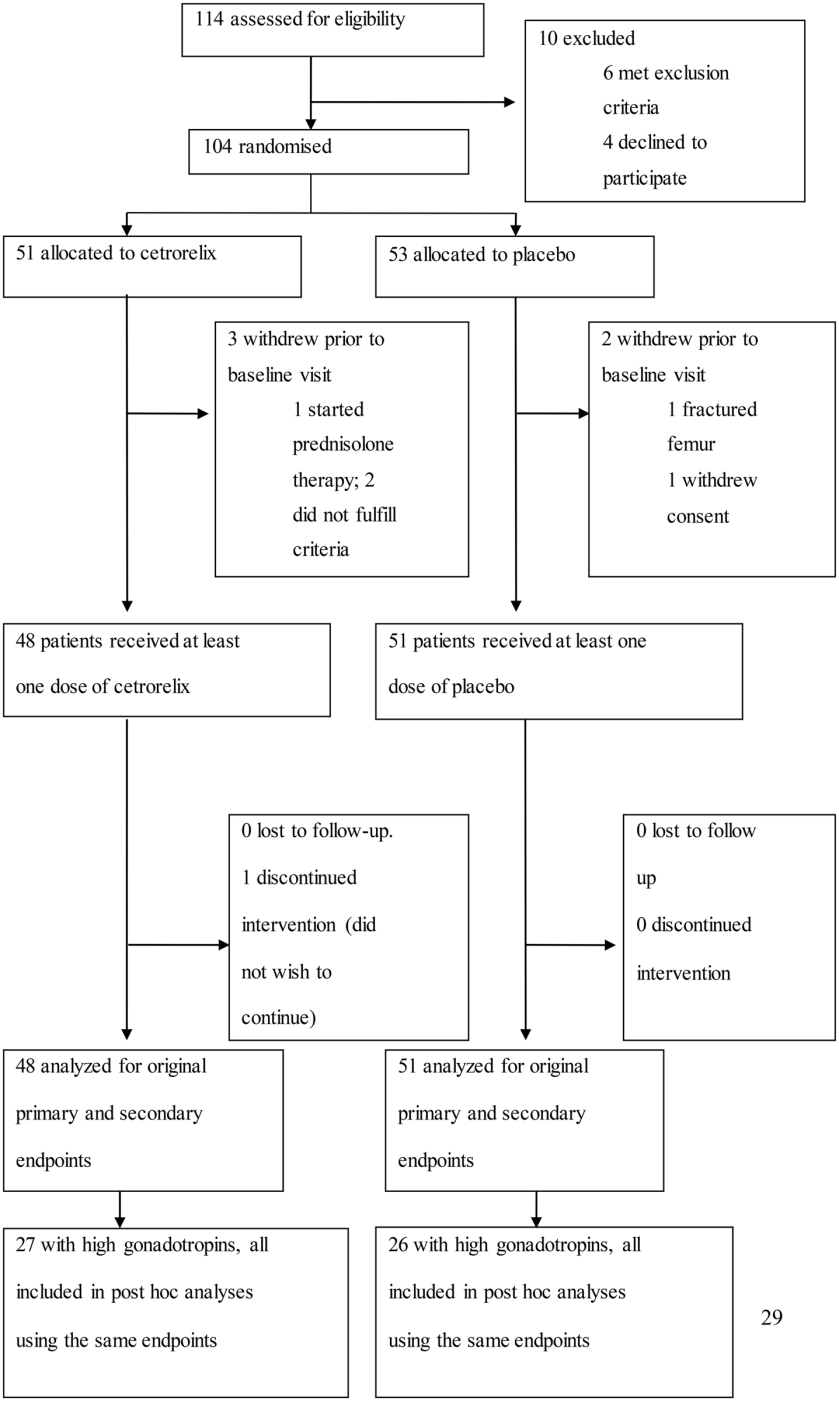
Trial Flow Chart.

Patient’s visits were performed between August 1, 2008 and June 11, 2011. As expected, compared to the whole AGRA group, patients with high gonadotropin levels were all postmenopausal females except for one male who also had high gonadotropin levels. Except for this, there were no significant differences in baseline variables or concomitant therapy, between any of the four groups (Table 1). For the remainder of the article, when referring to patients in the cetrorelix and placebo groups, we refer to the 53 patients with high gonadotropin levels (Fig 1) unless otherwise specified.

**Table 1:** Baseline Characteristics of the Participants*.

	CetrorelixAll N = 48	CetrorelixHigh Gonadotropins N = 27	Placebo All N = 51	Placebo High Gonadotropins N = 26
**DEMOGRAPHICS**				
Age, years	54∙9 ±11∙4	58.9 ± 8.3	55∙0 ±11∙7	59.8 ±8.8
Female ― no. (%)	35 (73)	26 (96)	36 (71)	26 (100)
Disease duration, years	11∙5 ±10∙6	12.9 ±11.6	12∙0 ±12∙9	15.6 ±15.5
Anti-CCP† antibody positive ― no. (%)	28 (58)	15 (56)	35 (69)	15(58)
Current smoker ―no. (%)	13 (27)	14 (52)	20 (39)	12 (46)
**CLINICAL AND LABORATORY MEASURES**				
DAS28-CRP‡	5∙0 ±1.0	5.0 ±1.0	5∙2 ±1.0	5.2 ±0.9
LH§ (IU/L)	20∙4 ±16∙3	32.0 ±12.4	19∙6 ±16∙3	34.2 ±9.8
FSH¶ (IU/L)	36∙9 ±30∙0	59.5 ±19.5	32∙9 ±31∙1	60.4 ±19.8
C-reactive protein (mg/L)	18∙9 ±24∙5	23.0±31.3	17∙3±22∙5	20.6±26.6
ESR‖ (mm/h)	22∙0 ±18.3	21.8 ±20.2	25∙8 ±27.0	29.7±30.4
Cortisol (nmol/L)	392 ±151	404 ±158	401 ±200	410 ±213
**CURRENT MEDICATION**				
None ― no. (%)	11 (23)	8 (30)	12 (24)	4 (15)
Stable NSAIDs** ―no. (%)	9 (19)	5 (19)	14 (27)	6 (23)
Stable prednisolone ≤ 7·5mg ―no. (%)	24 (50)	13 (48)	22 (43)	12 (46)
Stable DMARDs†† ― no. (%)	19 (40)	9 (33)	27 (53)	15 (57)
MTX	16 (33)	8 (30)	17 (33)	10 (38)
LEF	2 (4)	0	2 (4)	2 (8)
SSZ	0	0	1 (2)	0
HCQ	1 (2)	1 (4)	2 (4)	2 (8)
MTX + SLZ	0	0	3 (6)	1 (4)
MTX, SSZ + HCQ	0	0	2 (4)	0
**PREVIOUS FAILURE TO DMARDS OR BIOLOGIC THERAPY** ‡‡				
Previous failure with any number of DMARDs ―no.(%)	40 (83)	24 (89)	45 (88)	24 (92)
1 previous DMARD	13 (27)	8 (30)	13 (25)	7 (27)
2 previous DMARDs	10 (21)	6 (22)	9 (18)	4 (15)
≥3 previous DMARDs	17 (35)	10 (37)	23 (45)	13 (50)
Previous failure with any number of biologics―no. (%)	21 (44)	12 (44)	23 (45)	11 (42)
1 previous biologic	9 (19)	6 (22)	9 (18)	4 (15)
2 previous biologics	6 (13)	2 (7)	3 (6)	2 (8)
≥3 previous biologics	6 (13)	4 (15)	11(22)	5 (19)

* Plus—minus values are means ±SD. There were no significant differences between groups in baseline characteristics, except for age and sex.

^†^ CCP denotes Cyclic Citrullinated Peptide.

^‡^ DAS28-CRP Disease Activity Score for 28-joint counts based on C-reactive protein (in which scores range from 2 to 9, with higher scores indicating more disease activity).

^§^ LH denotes Luteinizing Hormone.

^¶^ FSH denotes Follicle-Stimulating Hormone, Based on detectable FSH values <256 IU/L; N = 48 (cetrorelix), N = 50 (placebo).

ESR denotes Erythrocyte Sedimentation Rate.

** NSAIDs denotes Nonsteroidal Anti-Inflammatory Drugs.

^††^ DMARDs denotes Disease-Modifying Anti-Rheumatic Drugs; MTX Methotrexate; SSZ Sulfasalazine; HCQ Hydroxychloroquine; and LEF Leflunomide.

^‡‡^ Previous failure includes inefficacy or intolerability.

### DAS28-CRP

DAS28-CRP decreased with cetrorelix by day 5 (−1.0; 95% CI -1.3 to -0.67) compared with placebo (−0∙40; 95% CI -0.71 to -0.19); baseline-adjusted between-group difference 0∙53, 95% CI 0.13 to 0.93; P = 0.010.

**Fig 2:**
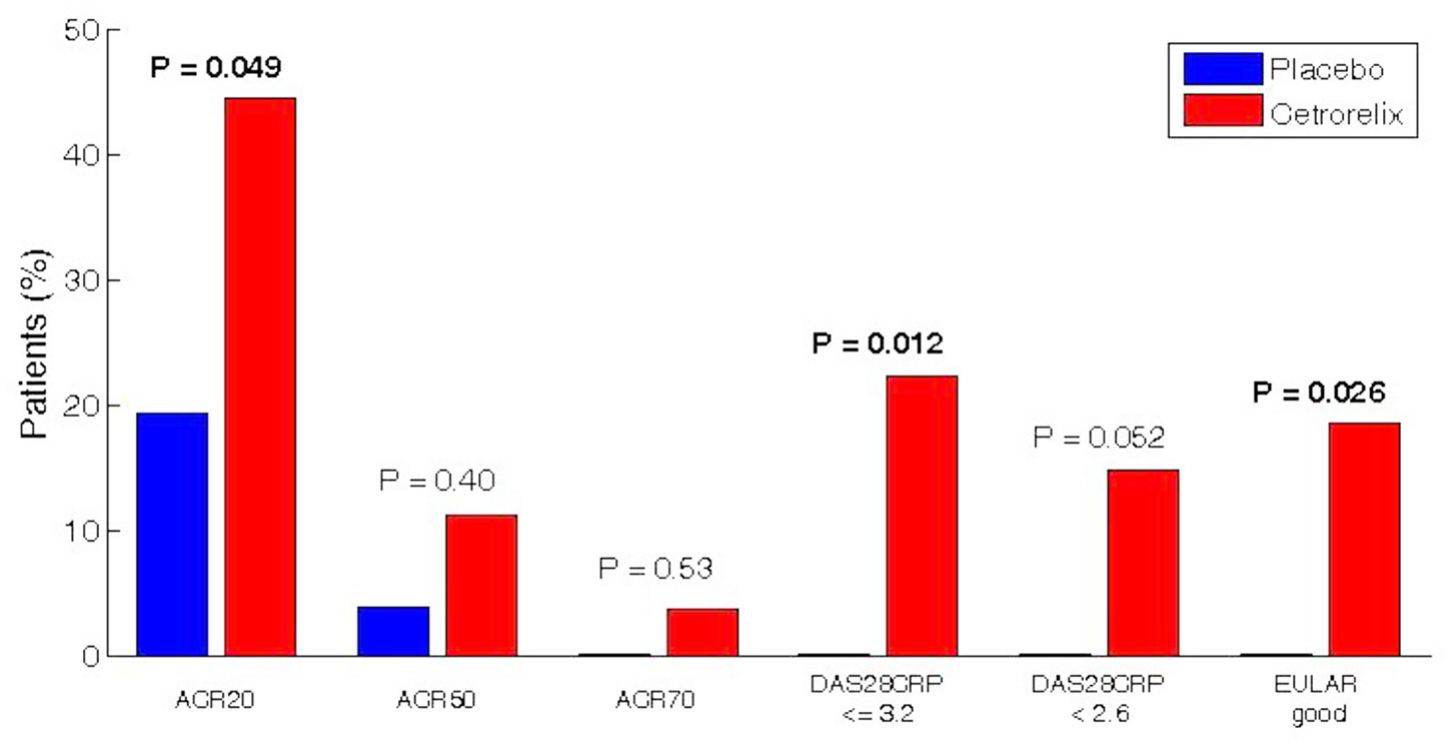
Efficacy Variables by Day 5 Between Groups. Fig 2 shows the percentage of patients achieving at least a 20%, 50%, 70% improvement in the American College of Rheumatology scale (ACR20, ACR50, and ACR70 respectively), the percentage of patients achieving ≤ 3.2 on the Disease Activity Score for 28-joint counts, based on C-reactive protein ([DAS28-CRP ≤ 3.2], in which scores range from 2 to 9, with higher scores indicating more disease activity), the percentage of patients achieving DAS28-CRP<2.6, European League Against Rheumatism ‘Good-Responses’ (EULAR good). All patients, N = 53, are included in these intention-to-treat analyses. There were no significant differences in disease activity measured by DAS28-CRP at baseline between groups.

More patients achieved DAS28-CRP≤3.2 (22% vs. 0%, P = 0.012) and DAS28-CRP<2.6 (15% vs. 0%; P = 0.052) by day 5 with cetrorelix, compared with placebo (Fig 2).

Among patients in both the cetrorelix and placebo groups combined, change in DAS28-CRP was associated with change in LH (Spearman-rho = 0.33; 95% CI 0.065 to 0.56; P = 0.016) and FSH (rho = 0.32; 95% CI 0.046 to 0.55; P = 0.023) but not with change in cortisol, oestradiol or testosterone.

### ACR and EULAR Responses

By day 5, more patients achieved ACR20 responses (44% vs.19%; P = 0.049) and EULAR ‘Good-Reponses’ (19% vs. 0%, P = 0.026) with cetrorelix, compared with placebo (Fig 2). More patients reached ACR50 and ACR70 responses with cetrorelix, although numbers were too small for valid conclusions.

### CRP

**Fig 3:**
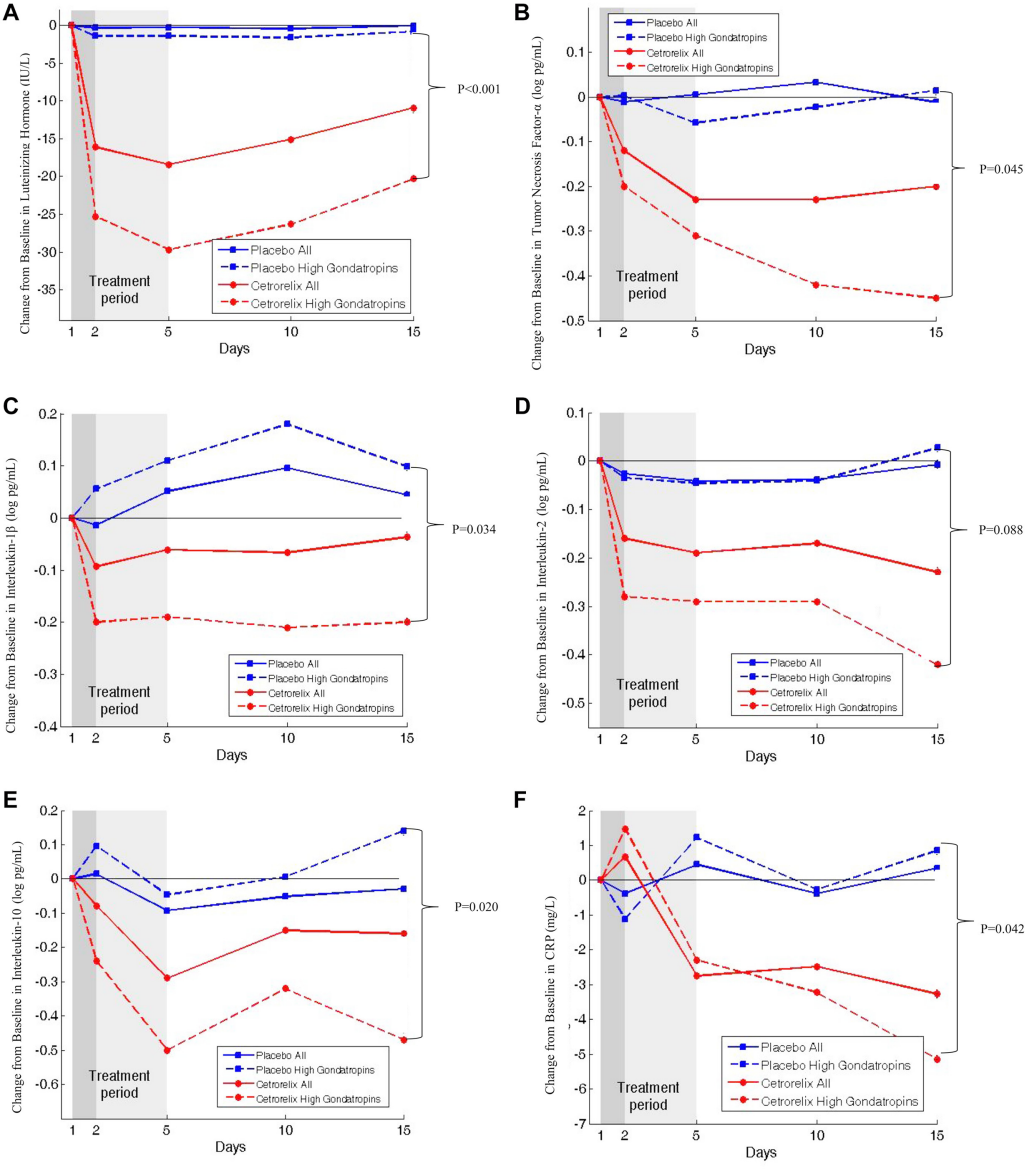
Change in Hormonal and Immunological Variables from Baseline. P values represent the baseline-adjusted between-group difference using analysis-of-covariance between the high-gonadotropin cetrorelix group (N = 27) and high-gonadotropin placebo group (N = 26) on day 15. There were no significant differences between the high-gonadotropin groups at baseline, day 2, day 5, or day 10; except for luteinizing hormone, which was significantly reduced in the cetrorelix groups compared to placebo groups already by day 2 onwards. Panels A-F show the change from baseline in luteinizing hormone, tumor necrosis factor-α, interleukin-1β, interleukin-2, interleukin-10, and C-reactive protein respectively. A: Change from Baseline in Luteinizing Hormone Between Groups. B: Change from Baseline in Tumor Necrosis Factor-α Between Groups. C: Change from Baseline in Interleukin-1β Between Groups. D: Change from Baseline in Interleukin-2 Between Groups. E: Change from Baseline in Interleukin-10 Between Groups. F: Change from Baseline in C-Reactive Protein (CRP) Between Groups.

CRP (mg/L) decreased with cetrorelix (-5.1), compared with an increase with placebo (0.85); between-group difference 5.45; 95% CI 0.22 to 10.7; P = 0.042 by day 15 (Fig 3A).

### Cytokines

TNF-α (log pg/mL) significantly decreased with cetrorelix (-0.45), compared with an increase with placebo (+0.02) by day 15 (P = 0.045). Interleukin-1β and interleukin-10 also significantly decreased with cetrorelix, compared with placebo (P = 0.034 and P = 0.020), and interleukin-2 decreased non-significantly with cetrorelix, compared with placebo (P = 0.088) by day 15 (Table 2; Fig 3B–3E).

**Table 2:** Cytokine Levels at Baseline and Final Visit*.

Cytokine (log pg/mL)	Baseline Day 1†	Final Day 15	Change from baseline by Day 15	Baseline-Adjusted Between-Group Difference, 95% CI
TNF-α§ Cetrorelix N = 27	0.46±2.3	0.01±2.1	-0.45	**0.49** ‡
TNF-α Placebo N = 26	0.97±3.0	0.99±3.1	+0.02	**(0.01, 0.96)**
IL¶-1β Cetrorelix N = 27	-1.48±1.5	-1.68±1.4	-0.20	**0.30** ‡
IL-1β Placebo N = 26	-1.21±2.4	-1.11±2.4	+0.10	**(0.02, 0.59)**
IL-2 Cetrorelix N = 27	-2.97±3.2	-3.39±2.9	-0.42	**0.51**
IL-2 Placebo N = 26	-2.32±3.7	-2.29±3.7	+0.02	**(-1.09, 0.08)**
IL-10 Cetrorelix N = 27	0.33±2.1	-0.13±2.1	-0.40	**0.59** ‡
IL-10 Placebo N = 26	0.16±2.5	0.29±2.5	+0.13	**(0.10, 1.08)**

* Plus—minus values are means ±SD; Intention-to-treat analyses of cytokine levels between high-gonadotropin groups.

^†^ There were no significant differences at baseline between groups in any cytokine.

^‡^ P<0.05 between group difference.

^§^ TNF-α denotes tumor necrosis factor-α.

^¶^ IL denotes interleukin.

Notably with cetrorelix, the reduction of LH (a surrogate marker for GnRH) and all cytokines was greatest during the first two days when the highest doses of cetrorelix were given (Fig 3).

### Onset and Offset

Although gonadotropin levels were decreased with cetrorelix already by day 2, there were no significant changes in clinical endpoints compared with placebo, until maximum gonadotropin suppression by day 5. As expected by day 10, gonadotropins increased after treatment withdrawal, increasing further towards baseline by day 15 (Fig 3A). The same trend was observed with DAS28-CRP and secondary endpoints. Although this suggested there was a rapid offset effect, interleukin-1β, interleukin-2 and interleukin-10 remained decreased, and TNF-α and CRP appeared to continue to decrease despite treatment withdrawal.

### Adverse Events

Adverse events occurred in similar frequencies in both groups, and were mild (Table 3).

**Table 3:** All Adverse Events during the Study Period in the High-Gonadotropin Cetrorelix and Placebo Groups.

Event*	Cetrorelix, N = 27	Placebo, N = 26
Headache—no. %	2 (7.4)	3 (11.5)
Injection site discomfort—no. %	2 (7.4)	0
Nausea—no. %	0	2 (7.7)

* There were no significant between-group differences.

## Discussion

This novel study indicates that short-term treatment with a GnRH-antagonist might have rapid disease ameliorating effects in RA patients with high gonadotropin levels. The original primary endpoint and several original key secondary endpoints were met. It is notable that a significantly greater proportion of patients achieved a DAS28-CRP≤3.2 (22% vs. 0%) and a EULAR ‘Good-Response’ (19% vs. 0%) on cetrorelix compared to placebo by day 5. There was also a trend towards a greater proportion of patients achieving DAS28-CRP<2.6 on cetrorelix compared to placebo (15% vs. 0%). Importantly, cetrorelix treatment resulted in significant improvements of objective biochemical endpoints, CRP, TNF-α, interleukin-1β and interleukin-10.

These results support the original trial with the entire AGRA group, where, although the improvement in DAS28-CRP with cetrorelix compared with placebo was not significant (P = 0.090), significantly more patients on cetrorelix achieved an ACR20 and DAS28-CRP<2.6, and TNF-α significantly decreased with cetrorelix, compared with placebo [16]. Compared to placebo, cetrorelix showed greater reductions of DAS28-CRP and cytokines in the high gonadotropin groups (N = 53) versus the rest of the AGRA population (N = 46) (Fig 3B–3E, supported by tests of interaction in the supplementary appendix, S1 Appendix). Thus, these findings support the biologically plausible hypothesis that GnRH-antagonism has a strong anti-inflammatory effect in RA patients with high gonadotropins.

GnRH antagonism was safe in our study; however the long-term safety of GnRH antagonist treatment in RA requires further investigation. As long-term GnRH antagonists in prostate cancer have a good safety profile, they seem to be attractive drugs for further investigation.

It is not known whether the cytokine reductions due to cetrorelix treatment might be mediated directly through GnRH or indirectly via downstream hormones such as gonadotropins. Cetrorelix has been shown to directly decrease pro-inflammatory cytokine gene expression through GnRH receptors in rats [25], and myeloma cells [26]. Therefore, it is not unlikely that cetrorelix reduces RA activity by down-regulating proinflammatory cytokine secretion via a direct effect on GnRH receptors on immune cells. The simultaneous reduction of cytokines, CRP, and disease activity in our study, might be explained, at least partially, through GnRH-receptor blockade on T cells. Peripheral T cells can secrete immunoactive GnRH, which acts upon these T cells through GnRH receptors, stimulating T-cell proliferation and maturation [7–12]. Another explanation may be through effects of GnRH on B cells [27].

Cetrorelix significantly reduced gonadotropins, oestradiol, testosterone and cortisol compared to placebo. DAS28-CRP correlated positively with gonadotropins, but not with oestradiol, testosterone or cortisol. Thus, it is more likely that the observed anti-inflammatory effect is mediated by GnRH and/or gonadotropin reduction, rather than oestradiol, testosterone or cortisol reduction. These findings are supported by our earlier longitudinal case-control study, where changes in gonadotropins, but not oestradiol, testosterone, cortisol or prolactin, positively correlated with changes in proinflammatory cytokines and disease activity markers in RA patients, and not in controls [23]. Furthermore, it has been demonstrated that RA flares are associated with gonadotropin elevations, but not with prolactin, cortisol or testosterone variations [28].

Oral contraceptives, hormone replacement therapy or testosterone therapy may reduce the activity of GnRH and gonadotropins due to negative feedback. The amount of HPG axis suppression is variable due to hormone formulation, dose, and concentration. This may help to explain the mixed results from trials involving oestrogen or testosterone [29]. Females are more susceptible than males to GnRH and gonadotropin increases, which are also more profound, due to the menopausal transition, postpartum, and anti-oestrogen therapy. Thus, our findings might help to explain why females are not only more susceptible to RA, but also have more severe disease than males.

The advantage of our post hoc analyses is that they are based on predefined analyses for the entire AGRA population, and are clinically relevant. The results are considered reliable as they are in accordance with the results of the original AGRA trial. Both the original primary and key secondary endpoints were met despite performing analyses in a proof-of-concept trial with patients of longstanding disease, of whom 89% had previously failed disease-modifying antirheumatic drugs or 45% failed biologic therapy or failed both. Such baseline characteristics usually decrease beneficial responses to therapy [30]. There was a trend towards slightly higher disease activity in controls (e.g. DAS28-CRP 5.2 vs. 5.0); although CRP was slightly lower in controls. It is not likely that this has substantially influenced our results as all analyses are baseline-adjusted and there were no significant differences in any outcome measures at baseline. Furthermore, one could speculate that a greater reduction in disease activity would be more easily apparent in patients with higher disease activity [30]. Inclusion criteria required that any patients on concomitant therapy had to be on long-term stable doses prior to study start. A varied concomitant DMARD background allowed the possibility of drug interactions that could confound our results. We believe it is unlikely that our results are confounded by this because baseline concomitant therapy was not significantly different between groups. A multicentre trial would have been required to recruit RA patients to a homogenous DMARD background, which was not feasible without proof-of-concept data. However, future studies should test GnRH antagonism in RA patients with homogenous background DMARD use. We recognize that this study was short. Nonetheless, the study’s design enabled the demonstration of rapid changes in clinical and biochemical parameters.

Our results suggest that GnRH, and/or gonadotropins are important in RA disease perpetuation. The data indicate a need for further studies examining GnRH-antagonists in RA, and possibly other autoimmune diseases, particularly in postmenopausal females and others with high gonadotropin levels.

## Supporting Information

S1 Appendix (DOCX)

S1 CONSORT Checklist CONSORT Checklist. (DOCX)

S1 Protocol Trial protocol. (PDF)

## References

[1] Pikwer M, Bergstrom U, Nilsson JA, Jacobsson L, Turesson C. Early menopause is an independent risk factor of rheumatoid arthritis. Ann Rheum Dis. 2012;71: 378–81. 21972241 10.1136/ard.2011.200059

[2] Wallenius M, Skomsvoll JF, Irgens LM, Salvesen KÅ, Koldingsnes W, Mikkelsen K, et al Postpartum onset of rheumatoid arthritis and other chronic arthritides: results from a patient register linked to a medical birth registry. Ann Rheum Dis. 2010;69: 332–6. 10.1136/ard.2009.115964 19717397

[3] Tan AL, Emery P. Role of oestrogen in the development of joint symptoms? Lancet Oncol. 2008;9: 817–8. 10.1016/S1470-2045(08)70217-1 18760239

[4] Merlino LA, Cerhan JR, Criswell LA, Mikuls TR, Saag KG. Estrogen and other female reproductive risk factors are not strongly associated with the development of rheumatoid arthritis in elderly women. Semin Arthritis Rheum. 2003;33: 72–82. 14625816 10.1016/s0049-0172(03)00084-2

[5] de Man YA, Dolhain RJ, van de Geijn FE, Willemsen SP, Hazes JM. Disease activity of rheumatoid arthritis during pregnancy: results from a nationwide prospective study. Arthritis Rheum. 2008;59: 1241–8. 10.1002/art.24003 18759316

[6] Kjeldsen-Kragh J, Haugen M, Borchgrevink CF, Laerum E, Eek M, Mowinkel P, et al Controlled trial of fasting and one-year vegetarian diet in rheumatoid arthritis. Lancet. 1991;338: 899–902. 1681264 10.1016/0140-6736(91)91770-u

[7] Chen A, Ganor Y, Rahimpour S, Ben-Aroya N, Koch Y, Levite M. The neuropeptides GnRH-II and GnRH-1 are produced by human T cells and trigger laminin receptor gene expression, adhesion, chemotaxis and homing to specific organs. Nat Med. 2002;8: 1421–6. 12447356 10.1038/nm1202-801

[8] Azad N, La Paglia N, Jurgens KA, Kirsteins L, Emanuele NV, Kelley MR, et al Immunoactivation enhances the concentration of luteinizing hormone-releasing hormone peptide and its gene expression in human peripheral T-lymphocytes. Endocrinology. 1993;133: 215–23. 8319570 10.1210/endo.133.1.8319570

[9] Morale MC, Batticane N, Bartoloni G, Guarcello V, Farinella Z, Galasso MG, et al Blockade of central and peripheral luteinizing hormone-releasing hormone (LHRH) receptors in neonatal rats with a potent LHRH-antagonist inhibits the morphofunctional development of the thymus and maturation of the cell-mediated and humoral immune responses. Endocrinology. 1991;128: 1073–85. 1846575 10.1210/endo-128-2-1073

[10] Chen HF, Jeung EB, Stephenson M, Leung PC. Human peripheral blood mononuclear cells express gonadotropin-releasing hormone (GnRH), GnRH receptor, and interleukin-2 receptor γ-chain messenger ribonucleic acids that are regulated by GnRH in vitro. J Clin Endocrinol Metab. 1999;84: 743–50. 10022447 10.1210/jcem.84.2.5440

[11] Batticane N, Morale MC, Gallo F, Farinella Z, Marchetti B. Luteinizing hormone-releasing hormone signaling at the lymphocyte involves stimulation of interleukin-2 receptor expression. Endocrinology. 1991;129: 277–86. 2055189 10.1210/endo-129-1-277

[12] Tanriverdi F, Silveira LF, MacColl GS, Bouloux PM. The hypothalamic-pituitary-gonadal axis: immune function and autoimmunity. J Endocrinol. 2003;176: 293–304. 12630914 10.1677/joe.0.1760293

[13] Sabharwal P, Varma S, Malarkey WB. Human thymocytes secrete luteinizing hormone: an autocrine regulator of T-cell proliferation. Biochem and Biophys Res Commun. 1992;187: 1187–92. 1530613 10.1016/0006-291x(92)91322-h

[14] Iqbal J, Sun L, Kumar TR, Blair HC, Zaidi M. Follicle-stimulating hormone stimulates TNF production from immune cells to enhance osteoblast and osteoclast formation. Proc Natl Acad Sci U S A. 2006;103: 14925–30. 17003115 10.1073/pnas.0606805103 PMC1595452

[15] Pope J, Joneja M, Hong P. Anti-androgen treatment of prostatic carcinoma may be a risk factor for development of rheumatoid arthritis. J Rheumatol. 2002;29: 2459–62. 12415609

[16] Kass AS, Forre OT, Fagerland MW, Gulseth HC, Torjesen PA, Hollan I. Short-term treatment with a gonadotropin-releasing hormone antagonist, cetrorelix, in rheumatoid arthritis (AGRA): a randomized, double-blind, placebo-controlled study. Scand J Rheumatol. 2014;43: 22–7. 10.3109/03009742.2013.825007 24182325 PMC3913106

[17] Englund M, Joud A, Geborek P, Felson DT, Jacobsson LT, Petersson IF. Prevalence and incidence of rheumatoid arthritis in southern Sweden 2008 and their relation to prescribed biologics. Rheumatology. 2010;49: 1563–9. 10.1093/rheumatology/keq127 20444855

[18] Sokka T, Toloza S, Cutolo M, Kautiainen H, Makinen H, Gogus F, et al Women, men and rheumatoid arthritis; analyses of disease activity, disease characteristics, and treatments in the QUEST-RA study. Arthritis Res Ther. 2009;11: R7 19144159 10.1186/ar2591 PMC2688237

[19] Arnett FC, Edworthy SM, Bloch DA, McShane DJ, Fries JF, Cooper NS, et al The American Rheumatism Association 1987 revised criteria for the classification of rheumatoid arthritis. Arthritis Rheum. 1988;31: 315–24. 3358796 10.1002/art.1780310302

[20] Wells G, Becker JC, Teng J, Dougados M, Schiff M, Smolen J, et al Validation of the 28-joint Disease Activity Score (DAS28) and European League Against Rheumatism response criteria based on c-reactive protein against disease progression in patients with rheumatoid arthritis, and comparison with the DAS28 based on erythrocyte sedimentation rate. Ann Rheum Dis. 2009;68: 954–60. 10.1136/ard.2007.084459 18490431 PMC2674547

[21] Felson DT, Andersson JJ, Boers M, Bombardier C, Furst D, Goldsmith C, et al American College of Rheumatology: preliminary definition of improvement in rheumatoid arthritis. Arthritis Rheum. 1995;38: 727–35. 7779114 10.1002/art.1780380602

[22] van Gestel AM, Prevoo ML, van’t Hof MA, van Rijswijk MH, van de Putte LB, van Riel PL. Development and validation of the European League Against Rheumatism response criteria for rheumatoid arthritis: comparison with the preliminary American College of Rheumatology and the World Health Organization/International League Against Rheumatism Criteria. Arthritis Rheum. 1996;39: 34–40. 8546736 10.1002/art.1780390105

[23] Kass AS, Lea RE, Torjesen PA, Gulseth, Forre OT. The association of luteinizing hormone and follicle-stimulating hormone with cytokines and markers of disease activity in rheumatoid arthritis: a case-control study. Scand J Rheumatol. 2010;39: 109–17. 10.3109/03009740903270607 20337546

[24] Lydersen S, Fagerland MW, Laake P. Recommended tests for association in 2 x 2 tables. Stat Med. 2009;28: 1159–75. 10.1002/sim.3531 19170020

[25] Rick FG, Schally AV, Block NL, Halmos G, Perez R, Fernandez JB, et al LHRH antagonist cetrorelix reduces prostate size and gene expression of proinflammatory cytokines and growth factors in a rat model of benign prostatic hyperplasia. Prostate. 2011;71: 736–47. 10.1002/pros.21289 20945403

[26] Wen J, Feng Y, Bjorklund CC, Wang M, Orlowski RZ, Shi ZZ, et al Luteinizing hormone-releasing hormone (LHRH)-I antagonist cetrorelix inhibits myeloma growth in vitro and in vivo. Mol Cancer Ther. 2011;10: 148–58. 21062912 10.1158/1535-7163.MCT-10-0829

[27] Ansari MA, Dhar M, Spieker S, Bakht N, Rahman AM, Moore WV, et al Modulation of diabetes with gonadotropin-releasing hormone antagonists in the nonobese mouse model of autoimmune diabetes. Endocrinology. 2004;145: 337–42. 12959992 10.1210/en.2003-0512

[28] Gordon D, Beastall GH, Thomson JA, Sturrock RD. Prolonged hypogonadism in male patients with rheumatoid arthritis during flares in disease activity. Br J Rheumatol. 1988;27: 440–44. 3144408 10.1093/rheumatology/27.6.440

[29] Cutolo M. Sex hormone adjuvant therapy in rheumatoid arthritis. Rheum Dis Clin North Am. 2000;26: 881–95. 11084949 10.1016/s0889-857x(05)70174-5

[30] Andersson JJ, Wells G, Verhoeven AC, Felson DT. Factors predicting response to treatment in rheumatoid arthritis: the importance of disease duration. Arthritis Rheum. 2000;43: 22–9. 10643696 10.1002/1529-0131(200001)43:1<22::AID-ANR4>3.0.CO;2-9

