# Supplementary material for: Rapid Anti-Inflammatory Effects of Gonadotropin-Releasing Hormone Antagonism in Rheumatoid Arthritis Patients with High Gonadotropin Levels in the AGRA Trial
Source: PLoS One. 2015 Oct 13;10(10):e0139439. doi: 10.1371/journal.pone.0139439 (PMC4603957; doi:10.1371/journal.pone.0139439)
Supplement: S1 Appendix — (DOCX) [file pone.0139439.s001.docx]

**Supplementary Appendix**

This appendix has been provided by the authors to give readers additional information about their work.

Supplement to: Kåss A, Hollan I, Fagerland MW, Gulseth HC, Torjesen PA, and Førre ØT. Rapid anti-inflammatory effects of gonadotropin-releasing hormone antagonism in rheumatoid arthritis patients with high gonadotropin levels in the AGRA trial.

**Supplementary Methods**

**Inclusion criteria**

- Age ≥ 18 years.
- Fulfilment of the revised 1987 American College of Rheumatology (ACR) criteria for the classification of RA.
- Moderate or severely active RA, defined as a DAS28>3.2 and at least two of the following criteria: ≥ 6 painful joints, ≥ 3 swollen joints, ESR ≥ 20mm/h, and a C-reactive protein ≥ 10mg/L).
- Prednisolone ≤ 7.5mg/day permitted if stable for at least 4 weeks prior to baseline, NSAIDs permitted if stable for at least 2 weeks prior to baseline; disease-modifying anti-rheumatic drugs (DMARDs) permitted if stable for at least 8 weeks prior to baseline.
- A negative pregnancy test for women of childbearing potential prior to start of treatment.
- Use of reliable method of contraception e.g. intra-uterine devices or sheaths by all sexually active patients. If the patient were abstinent, they had to agree to stay abstinent or use reliable contraception if sexual activity commences.
- Menstruating women could enter the study in the early follicular phase of their menstrual cycle only.
- Able and willing to give written informed consent and to comply with the requirements of the study protocol.

**Exclusion Criteria**

- History of positive HIV status.
- History of TB, histoplasmosis, or listeriosis.
- History of hormone-dependent cancers ever or history of non-hormone-dependent cancers within 5 years of screening visit.
- Tumour necrosis factor-α (TNF-α) inhibitor or other biological agents were not permitted during the trial or within 4 weeks prior to inclusion. (Infliximab/adalimumab was not permitted at least 3 months prior to inclusion; Rituximab was not permitted at least 6 months prior to inclusion).
- Persistent or recurrent infections or severe infections requiring hospitalization or treatment with iv antibiotics within 30 days, or oral antibiotics within 14 days prior to enrolment.
- Pregnancy or breast-feeding.
- Any treatment with hormone replacement therapy or oral contraception.
- Significant renal or hepatic impairment.
- Intramuscular, intra-articular or intravenous injections of corticosteroids were not permitted during the trial or within 4 weeks prior to inclusion.
- Vaccination with living vaccines were not allowed during the treatment

**Hormone Assays**

Non-competetive immunofluorometric assays were used for the quantitative determination of serum luteinizing hormone (LH) and follicle-stimulating hormone (FSH) (Dissociation Enhanced Lanthanide Fluoroimmunoassay [DELFIA] kit, Turku, Finland). A competitive immunofluorometric assay was used for the determination of serum oestradiol (DELFIA kit, Turku, Finland). A competitive radioimmunoassay was used for the quantitative determination of serum testosterone (Orion Diagnostica, Espoo, Finland). A competitive luminoimmunoassay was used for the quantitative determination of serum cortisol (Immulite 2000, California, USA).

**Supplementary Results**

**Cortisol**

Cortisol (nmol/L) significantly decreased with cetrorelix, compared with placebo by day 5 (between-group difference 53.1, 95% CI 10.5 to 95.8, P=0.016). This reduction was not associated with patients’ use of prednisolone. Furthermore, among patients who received cetrorelix, DAS28-CRP reduction was similar in prednisolone users (N=13) and non-users (N=14; P=0.73).

**Sex**

As almost all patients in the ‘high gonadotropin’ group were female, we wanted to explore the effect of sex on treatment response. In the whole group, N=99, the overall treatment effect was 0.34 (95% CI -0.021 to 0.69), P=0.065 when regressing the clinical outcome, DAS-CRP, on the treatment group by adjusting for sex.

**Tests of Interaction**

We performed tests of interaction to test whether the treatment difference in outcome measures depended on whether the patient had high gonadotropins or not. Results were statistically significant when testing for the interaction between treatment and gonadotropin level (‘high N=53’ vs. ‘remainder of AGRA population N=46’) for TNF-α (log pg/mL) P=0.049, interleukin-1β (log pg/mL) P=0.016, interleukin-2 (log pg/mL) P=0.010, and interleukin-10 (log pg/mL) P=0.028. Therefore, these interaction tests suggest GnRH-antagonism reduced cytokines more in patients with high gonadotropins (also shown in Fig 3B-E in the original manuscript) compared to the rest of the AGRA population. GnRH-antagonism also showed a trend towards a greater reduction of DAS28CRP, P=0.051, in patients with high gonadotropins compared to the rest of the AGRA population. The numbers of males in this study did not allow for proper testing of interaction between treatment and sex.
