# Supplementary material for: Rapid Anti-Inflammatory Effects of Gonadotropin-Releasing Hormone Antagonism in Rheumatoid Arthritis Patients with High Gonadotropin Levels in the AGRA Trial
Source: PLoS One. 2015 Oct 13;10(10):e0139439. doi: 10.1371/journal.pone.0139439 (PMC4603957; doi:10.1371/journal.pone.0139439)
Supplement: S1 CONSORT Checklist — (DOCX) [file pone.0139439.s002.docx]

CONSORT 2010 checklist

| Section/Topic | Item No. | Checklist Item | Page No. |
| --- | --- | --- | --- |
| Title and Abstract |  |  |  |
|  | 1a | Identification as a randomised trial in the title | If required, will be added |
|  | 1b | Structured summary of trial design, methods, results and conclusion (see CONSORT abstract checklist) | Abstract |
| Introduction |  |  |  |
| Background and objectives | 2a | Scientific background and explanation of rationale | P4-6 |
|  | 2b | Scientific obejectives or hypotheses | P5-6 (Further details in protocol) |
| Methods |  |  |  |
| Trial design | 3a | Description of trial design | P7-8 |
|  | 3b | Important changes to methods after trial commencement (such as eligibility criteria). With reasons | P 7 |
| Participants | 4a | Eligibility criteria for participants | P7+ Supplement |
|  | 4b | Settings and locations where the data were collected | P7 |
| Interventions | 5 | The interventions for each group with sufficient details to allow replication, including how and when they were actually administered | P8-9 |
| Outcomes | 6a | Completely defined pre-specified primary and secondary outcome measures, including how and when they were assessed | P9-11 |
|  | 6b | Any changes to trial outcomes after the trial commenced with reasons | - |
| Sample size | 7a | How sample size was determined | P10 |
|  | 7b | When applicable, explanation of any interim analyses and stopping guidelines | - Criteria for termination of trial in protocol. No planned interim analyses |
| Randomization |  |  |  |
| Sequence generation | 8a | Method used to generate the random allocation sequence | P8 |
|  | 8b | Type of randomization, details of any such restriction (such as blocking and block size) | P8 |
| Allocation concealment mechanism | 9 | Mechanism used to implement the random allocation sequence (such as sequentially numbered containers), describing any steps taken to conceal the sequence | P8-9 |
| Implementation | 10 | Who generated the random allocation sequence, who enrolled participants, and who assigned participants to interventions | P8 (Further details in protocol) |
| Blinding | 11a | If done, who was blinded after assignment o interventions (for example, participants, care providers, those assessing outcomes) and how. | P8-9 |
|  | 11b | If relevant, description of the similarity of interventions | P8 |
| Statistical methods | 12a | Statistical methods used to compare groups for primary and secondary outcomes | P9-11 |
|  | 12b | Methods for additional analyses, such as subgroup analyses and adjusted analyses | P9-11 |
| Results |  |  |  |
| Participant flow | 13a | For each group, the numbers of participants who were randomly assigned received intended treatment, and were analysed for the primary outcome | Figure 1 |
|  | 13b | For each group, losses and exclusions after randomization, together with reasons | Figure 1 |
| Recruitment | 14a | Dates defining the periods of recruitment and follow up | P12 |
|  | 14b | Why the trial ended or was stopped | - |
| Baseline data | 15 | A table showing baseline demographic and clinical characteristics for each group | Table 1 |
| Numbers analysed | 16 | For each group, number of participants (denominator) included in each analysis and whether the analysis was by original assigned groups | All tables |
| Outcomes and estimation | 17a | For each primary and secondary outcome, results for each group, and the estimated effect size and its precision (such as 95% CI) | Results in text/ tables. |
|  | 17b | For binary outcomes, presentation of both absolute and relative effect sizes is recommended | - |
| Ancillary analyses | 18 | Results of any other analyses performed including subgroup analyses and adjusted analyses, distinguishing prespecified from exploratory | These are post hoc analyses based on protocol-specified analyses from the original trial |
| Harms | 19 | All important harms or unintended effects in each group (for specific guidance see CONSORT for harms) | P14 and Table 3 |
| Discussion |  |  |  |
| Limitations | 20 | Trial limitations, addressing sources of potential bias, impreceision, and if relevant, multilplicity of analyses | P19 |
| Generalisability | 21 | Generalisability (external validity, applicability) of the trial findings | P18-19 |
| Interpretation | 22 | Interpretation consistent with results, balancing benefits and harms, and considering other relevant evidence | P15-19 |
| Other information |  |  |  |
| Registration | 23 | Registration and name of trial registry | P1+7 |
| Protocol | 24 | Where the full trial protocol can be accessed, if available | Eventually can be accessed online with published article |
| Funding | 25 | Sources of funding and other support (such as supply of drugs), role of funders | Disclosed online |
