# Supplementary material for: Rapid Anti-Inflammatory Effects of Gonadotropin-Releasing Hormone Antagonism in Rheumatoid Arthritis Patients with High Gonadotropin Levels in the AGRA Trial
Source: PLoS One. 2015 Oct 13;10(10):e0139439. doi: 10.1371/journal.pone.0139439 (PMC4603957; doi:10.1371/journal.pone.0139439)
Supplement: S1 Protocol — (PDF) [file pone.0139439.s003.pdf]

Protocol Title:

Antagonising Gonadotropin-Releasing Hormone in Rheumatoid Arthritis (AGRA)

EudraCT number: 2007-001260-74

Protocol Code Number: R1/05

Version and Date: 19, 12th March 2011

Principal Investigator (Sponsor):

Dr. Anita Kåss

Department of Rheumatology

Betanien Hospital

Bjørnstjerne Bjørnsonsgt 6

3722 Skien

Norway

Telephone: +47 900 34786 Fax: +47 35900779

Email Address: anita.kass@betanienhospital.no

Collaborators:

Dr.med Ivana Hollan

Lillehammer Hospital for Rheumatic Diseases

2609 Lillehammer

Norway

Professor Øystein Førre

Centre for Rheumatic Diseases

Oslo University Hospital

0424 Oslo

Norway

Dr. Peter Torjesen

Department of Endocrinology

Aker University Hospital

0514 Oslo

Norway

Dr Hans Christian Gulseth

Head of Department of Rheumatology

Betanien Hospital

3722 Skien

Norway

This document is confidential and must not be communicated to a third party or reproduced or quoted without written consent of the principal investigator.

Research Nurse Gunn Teigen Næss

Department of Rheumatology

Betanien Hospital

3722 Skien

Norway

Martha Colban

Head of Clinical Research Support

Oslo University Hospital

0424 Oslo

Norway

Dr Morten Wang Fagerland

Unit of Biostatistics and Epidemiology

Oslo University Hospital

0424 Oslo

Norway

Professor Tor Lea

Dept. of Chemistry, Biotechnology and Food Science

Norwegian University of Life Sciences

1432 Aas

Norway

This document is confidential and must not be communicated to a third party or reproduced or quoted without written consent of the principal investigator.

TABLE OF CONTENTS

1. List of Abbreviations 7

2. Background 8

   2.1. Rationale of study 8

   2.2. Hypothesis 8

   2.3. Summary of findings from studies relevant to the trial 9

   2.4. Name and Description of Investigational Product 10

   2.5. Previous Studies Investigating Safety 11

      2.5.1. In Males 11

      2.5.2. In Females 11

   2.6. Known Risks 12

3. Aim 12

   3.1. Primary Aim 12

   3.2. Secondary Aims 13

4. Study Design 13

   4.1. Overview of study design 13

   4.2. Number of Patients/Assignments to Treatment Groups 14

   4.3. Patient Randomization and Enrolment 14

   4.4. Procedure for individual unblinding in association with an SAE 15

5. Study Population 15

   5.1. Target Population 15

   5.2. Inclusion Criteria 16

   5.3. Exclusion Criteria 17

6. Schedule of Assessments and Procedures 17

   6.1. Study Procedures 17

6.2. Informed Consent 18

6.3. Pregnancy Test 18

6.4. Screening Procedures 20

6.4.1. DAS28 Assessment 21

6.5. Medical History 21

6.6. Baseline Examination/Investigation 21

6.7. Study Assessments 22

6.7.1. Clinical Assessments 22

6.7.2. Physical Examination 23

6.7.3. Vital Signs 24

6.7.4. Laboratory Assessments 24

7. Treatment of Subjects 24

7.1.1. Cetrorelix Treatment and Rationale 24

7.1.2. Placebo Treatment 25

7.2. Concomitant Treatment 25

7.3. Rescue Medication 25

7.4. Administration of the Investigational Drug and Placebo 26

7.5. Blinding 26

8. Safety Issues 27

8.1. Adverse Events 27

8.2. Laboratory Test Abnormalities 29

8.3. Handling of Safety Parameters 29

8.3.1. Serious Adverse Events 29

8.3.2. Serious Adverse Event Reporting 30

8.3.3. Treatment and Follow-Up of Adverse Events 30

This document is confidential and must not be communicated to a third party or reproduced or quoted without written consent of the principal investigator.

8.3.4. Follow-up of Abnormal Laboratory Test Values 30

8.4. Discontinuation Criteria 30

9. Statistics 31

9.1. Sample Size 31

9.2. Analysis Populations 31

9.2.1. Safety Population 31

9.2.2. Intention To Treat (ITT) Population 32

9.2.3. Per Protocol Population 32

9.3. Statistical and Analysis Methods 32

9.3.1. Assessment of Treatment Response 33

9.3.2. Primary Endpoint and Analysis 34

9.3.3. Secondary Endpoints and Analyses 34

9.4. Missing Data 37

9.5. Safety Data Analyses 38

9.6. Interim Analysis 38

9.7. Replacement Policy 38

9.8. Criteria for Termination of the Trial 38

10. Monitoring and Quality Assurance 39

11. Ethics 40

11.1. Ethical Conduct of Trial 40

11.2. Patient Information and Consent 40

11.3. Withholding Treatment 41

12. Data Handling and Record Keeping 41

13. Publication Policy 42

14. References 43

This document is confidential and must not be communicated to a third party or reproduced or quoted without written consent of the principal investigator.

1 LIST OF ABBREVIATIONS

ACR	American college of rheumatology
AE	Adverse event
BDMARD	Biological disease modifying anti-rheumatic drug
CCP	Cyclic citrullinated peptide antibody
CRF	Case report form
CRP	C-reactive protein
DAS28	Disease activity score with 28 joint count
DMARD	Disease modifying anti-rheumatic drug
EC	Ethics committee
ESR	Erythrocyte sedimentation rate
EULAR	European league against rheumatism
FSH	Follicle-stimulating hormone
GCP	Good Clinical Practice
GnRH	Gonadotropin-releasing hormone
HAQ	Health assessment questionnaire
HCG	Human chorionic gonadotropin
HIV	Human immunodeficiency virus
HPG	Hypothalamic-pituitary-gonadal
ICH	International Conference on Harmonisation
IL	Interleukin
IP	Interphalangeal
IVRS	Interactive voice response system
LH	Luteinising hormone
LHRH	Luteinising hormone-releasing hormone
MA	Medicines agency
MIP	Macrophage inflammatory protein
MCP	Metacarpophalangeal
MCP	Monocyte chemoattractant protein
mRNA	Messenger ribonucleic acid
MTX	Methotrexate
NSAID	Non-steroidal anti-inflammatory drug
PIP	Proximal interphalangeal
RA	Rheumatoid arthritis
REK	Regional ethics committee (Regional komité for medisinsk forskningsetikk)
SAE	Serious Adverse Events
s.c.	Subcutaneous
SD	Standard deviation
SJC	Swollen joint count
SLE	Systemic lupus erythematosus
SLV	Norwegian medicines agency (Statens legemiddelverk)
SUSAR	Suspected unexpected serious adverse reaction
TJC	Tender joint count
TNF	Tumour necrosis factor
VAS	Visual analogue scale

This document is confidential and must not be communicated to a third party or reproduced or quoted without written consent of the principal investigator.

2 BACKGROUND

2.1 Rationale of study

Rheumatoid arthritis (RA) affects up to 1 % of the world's population[1] and is associated with significant morbidity[2] and mortality[3]. It is a chronic inflammatory disease characterized by infiltrating leukocytes in the synovium, progressive cartilage destruction and bone erosion. Despite major advances in the treatment for RA, patients do not always qualify for, do not always tolerate, or experience an insufficient response to currently available therapy. This study investigates a novel method of treating RA and can aid in the understanding of hormones in RA.

2.2 Hypothesis

The aetiology of RA is thought to be genetically, environmentally and hormonally related. Hormonal mechanisms, contributing to RA pathophysiology are not fully understood. However, bidirectional mechanisms between the endocrine and immune systems have been described previously. [4,5,6,7]

RA tends to develop, flare or subside during changes in hypothalamic pituitary gonadal (HPG) axis function such as the menstrual cycle, pregnancy, postpartum, and the menopause. [8,9,10,11] All of these situations are associated with substantial changes in the secretion of hormones of the HPG axis which are regulated primarily by the hypothalamic hormone, gonadotropin-releasing hormone (GnRH). GnRH has been shown to be immunostimulatory, (see section 2.3) and perhaps GnRH contributes to changes in disease activity observed during such hormonal changes. Therefore it is possible that treatment with GnRH-antagonists may reduce RA activity.

This document is confidential and must not be communicated to a third party or reproduced or quoted without written consent of the principal investigator.

[redacted]

2.3 Summary of findings from studies that are relevant to the trial

GnRH receptor and GnRH mRNA expression has been identified in human peripheral lymphocytes suggesting a potential autocrine and/or paracrine effect of GnRH in immune system regulation.[12,13] Human peripheral T cells (CD4+, CD8+) and the leukaemic (Jurkat) cell line similar to T lymphocytes have been to shown to produce immunoactive and bioactive GnRH.[14,15] GnRH *agonists* have been reported to increase thymus weight in adult rats.[16] In contrast, GnRH *antagonists*, blocked thymocyte proliferative responses and decreased CD4+ percentages and thymus weights in neonatal rats.[17]

GnRH *antagonists* might also act on the immune system through B- lymphocytes, for example GnRH antagonists decreased B cell percentages and ameliorated murine lupus independently of gonadal sex hormone secretion in castrated mice, whereas GnRH *agonists* increased lupus disease severity in gonadectomised mice of both sexes.[18] GnRH may also act indirectly through changes in pituitary hormones (luteinising hormone [LH] and follicle-stimulating hormone [FSH]). Indeed in vitro studies have shown that both LH and FSH have a stimulatory effect on IL-2 induced stages of T-cell activation.[19] Furthermore, FSH directly stimulated tumour necrosis factor (TNF)-α production from bone marrow granulocytes and macrophages.[20] Notably, significant associations have been observed between percent

This document is confidential and must not be communicated to a third party or reproduced or quoted without written consent of the principal investigator.

changes from baseline, of gonadotropins (LH and FSH) and RA disease activity, and between gonadotropins and key cytokines (i.e. TNF- α) in RA patients.[21]

2.4 Name and Description of Investigational Product

There are several GnRH-antagonists available. The two most common GnRH-antagonists in clinical studies are cetrorelix and ganirelix. Cetrorelix was preferred as there is more clinical data available on cetrorelix, and cetrorelix has a higher suppressive rate per milligram of peptide than other antagonists.

GnRH-Antagonist:

'Cetrorelix'

Approved Name:

'Cetrorelix Acetate'

Brand Name:

'Cetrotide' ASTA Medica

Therapeutic Class:

'Gonadotropin-releasing hormone antagonist' Cetrorelix is a GnRH antagonist which competitively blocks the binding of GnRH to GnRH receptors. This results in a dose-dependent suppression of the release of gonadotropins i.e. LH and FSH.

Licensed Indications:

Prevention of premature ovulation in patients undergoing controlled ovarian stimulation followed by oocyte pick-up and assisted reproductive techniques.

Pharmacology:

Cetrorelix is a decapeptide and a potent GnRH antagonist. It competitively inhibits the secretion of LH and FSH from the pituitary gland. Female and male volunteer studies show that cetrorelix produces a dose-dependent suppression of FSH, LH and oestradiol that is

maintained throughout continuous treatment and is reversible after treatment discontinuation.[22,23,24,25,26] Cetrorelix and GnRH antagonists in general, have an advantage over GnRH agonists in that they lack the initial flare up of gonadotropins and therefore have a shorter treatment period.

2.5 Previous Studies Investigating Safety

2.5.1 Previous Studies Investigating Safety in Males

Cetrorelix has been tested in males,[27,28,29,30,31] as well as females. Behre et al administered 1.0, 2.0, and 5.0 mg of subcutaneous cetrorelix in 30 healthy males. LH and testosterone were suppressed; maximal suppression occurring 4-6 h after injection. Hormone levels returned to normal within 24 hours after the 1.0 and 2.0mg doses; and within 48 hours for the 5.0mg dose. The only side effect observed after the administration of cetrorelix was a transient local erythema at the injection site that disappeared with 30 minutes. No changes in vital signs, clinical chemistry, whole blood count, or urinalysis were found. GnRH-antagonists have been studied as potential treatments for prostate cancer, benign prostatic hypertrophy and male contraception.[32,33,34,35]

2.5.2 Previous Studies Investigating Safety in Pre-menopausal and Postmenopausal Females

Pre-menopausal and postmenopausal female volunteer studies show that cetrorelix produces a dose-dependent suppression of FSH, LH and oestradiol that is maintained throughout continuous treatment and is reversible after treatment discontinuation.[36,37,38,39,40] A single injection during the late follicular phase delays the LH surge, even in cases where the surge has already begun.[41] This data also showed that cetrorelix was well tolerated. Another study examined the effects of daily GnRH antagonist during the periovulatory period. Injections of

10mg/day on 2, 3 and 5 consecutive days were administered. In half of the patients, demise of the dominant follicle was observed and a new cycle was initiated. In the other half of the patients, ovulation was delayed but not inhibited.[42] Cetrorelix has also been administered to women in the late luteal phase which reduces the pace of follicular development. In general, whatever phase of the menstrual cycle, GnRH antagonists give an immediate receptor blockage of gonadotrophic cells, which causes an immediate , but reversible, subsequent decrease of LH, FSH and oestradiol secretion for approximately 1-4 days after a single dose. The weekly administration (i.e. in all phases of the menstrual cycle) of 3mg of cetrorelix in women with endometriosis was effective and safe.[43]

2.6 Known Risks

Common (>1/100) side effects are from the injection site, for example, irritation, itch or redness. Less common side effects are nausea and headache. Rare (<1/1000) side effects are intense itching on the skin and serious hypersensitivity reactions, including anaphylactic reactions.

3 AIM

3.1 Primary Aim

The primary aim of this study is to assess whether cetrorelix acetate therapy can give a beneficial clinical response (measured by the change from baseline in the disease activity score calculated with C-reactive protein levels [DAS28CRP]) in RA patients with moderate to severe disease activity, and therefore be of future therapeutic benefit. For comparison, it was considered necessary to include a placebo group.

This document is confidential and must not be communicated to a third party or reproduced or quoted without written consent of the principal investigator.

3.2 Secondary Aims

Secondary aims of this study include:

- To assess whether cetrorelix is safe in rheumatoid arthritis patients.
- To assess whether cetrorelix acetate can decrease acute phase reactants (C-reactive protein [CRP], erythrocyte sedimentation rate [ESR]), anti-cyclic citrullinated peptide (anti-CCP) antibody levels and cytokine levels in RA patients.
- To assess whether cetrorelix can give a beneficial clinical response using American College of Rheumatology (ACR) response criteria 'ACR20/50/70/(90)', and European League Against Rheumatism (EULAR) response criteria 'Good', 'Moderate', 'None', and the proportion of DAS28CRP low disease activity/remission.
- To assess whether cetrorelix can decrease disability (measured by the Health Assessment Questionnaire [HAQ] score).
- To gain an increased understanding of the role of GnRH in the pathogenesis of RA.

4 STUDY DESIGN

4.1 Overview of Study Design and Rationale for Study Design

This study is a proof-of-concept, double-blind, randomised, placebo-controlled design to assess the efficacy and safety of cetrorelix acetate in the treatment of RA patients.

A total of 98 patients with a diagnosis of RA fulfilling study eligibility criteria will be enrolled at Betanien Hospital, Skien, Norway. Study medications cetrorelix acetate

This document is confidential and must not be communicated to a third party or reproduced or quoted without written consent of the principal investigator.

(5mg/d for the first two days and 3mg/d for the following 3 days, in total 5 consecutive days of medication) or placebo sodium chloride solution 5ml/d for the first 2 days and 3ml/d for the following 3 days will be administered subcutaneously by the research nurse. Efficacy and safety measurements will be performed throughout the study from baseline to day 15. There is a screening period of 28 days.

As hypothalamic suppression of reproductive function has not been investigated in RA before, the intervention is limited to a short period. Previous studies suggest that after a threshold of approximately 3–5mg s.c., increasing the dose does not significantly increase the antagonistic effect of cetrorelix on GnRH receptors. However, repeated doses with short time intervals both increase *and* prolong the antagonistic effect of cetrorelix.

4.2 Number of Patients/Assignments to Treatment Groups

98 patients will be recruited into this study and will be randomised equally into the 2 intervention groups. Patients will be stratified by sex to avoid any random gender bias between the two groups. There will be no replacement of patients should a patient's treatment be discontinued for any reason.

4.3 Patient Randomisation and Enrolment

Patients will be evaluated for eligibility to ensure the criteria in section 5.2 and 5.3 are filled. All patients must sign the Informed Consent form prior to screening. A screening form will be filled for each patient. Eligible patients will be randomised, through computer generated allocation, prior to receiving any study medication; this is carried out by study personnel who are not involved with outcome assessments. Randomisation

This document is confidential and must not be communicated to a third party or reproduced or quoted without written consent of the principal investigator.

will be carried out in dynamic blocks (varying block sizes unknown to the study site), and stratified for sex. Once a patient has been through the screening process and has given informed consent for participation, the research nurse obtains randomisation information by calling an offsite central office. The patient will be provided with a unique medication number and randomisation number. As confirmation, the investigator will be provided with a posted verification of each patient's registration.

A patient's treatment assignment should only be unblinded when knowledge of the treatment is essential for the further management of the patient.

4.4 Procedure for individual unblinding in association with an SAE

The investigator, in consultation with the clinical team, will assess the need for unblinding where an SAE has occurred and the allocation code is required, in order to enable clinical treatments to be planned. The central randomisation service will then be contacted if necessary. The following details will be provided: caller details (own name, position, location), name of patient participating and study protocol number and identification. Upon unblinding the site personnel will record the participant withdrawal and allocation in the person's own clinical and trial notes along with appropriate clinical information.

5 STUDY POPULATION

5.1 Target Population

The target population for this study is patients with moderate to severe RA (defined as DAS28 ≥3.2). Subjects will be screened to determine if they meet all the inclusion criteria specified in section 5.2 and none of the exclusion criteria specified in section 5.3.

This document is confidential and must not be communicated to a third party or reproduced or quoted without written consent of the principal investigator.

5.2 Inclusion Criteria

A subject will be eligible for study participation if he/she meets the following criteria:

- Age ≥ 18 years
- Fulfilment of the revised 1987 American College of Rheumatology (ACR) criteria for the classification of RA.
- Moderate or severely active RA, defined as a DAS28 ≥3.2 and at least two of the following criteria: ≥ 6 painful joints, ≥ 3 swollen joints, ESR ≥ 20mm/h, and a C-reactive protein ≥ 10mg/L
- Prednisolone ≤ 7.5mg/day permitted if stable for at least 4 weeks prior to baseline, NSAIDs permitted if stable for at least 2 weeks prior to baseline; disease modifying anti-rheumatic drugs (DMARDs) permitted if stable for at least 8 weeks prior to baseline
- A negative pregnancy test for women of childbearing potential prior to start of treatment
- Use of reliable method of contraception e.g. intra-uterine devices or sheaths by all sexually active patients. If the patient is abstinent, they must agree to stay abstinent or use reliable contraception if sexual activity commences.
- Menstruating women may enter the study in the early follicular phase of their menstrual cycle only
- Able and willing to give written informed consent and to comply with the requirements of the study protocol

This document is confidential and must not be communicated to a third party or reproduced or quoted without written consent of the principal investigator.

5.3 Exclusion Criteria

A subject will be excluded from the study if he/she meets any of the following criteria:

- History of positive HIV status
- History of TB, histoplasmosis, or listeriosis,.
- History of hormone-dependent cancers ever or history of non-hormone-dependent cancers within 5 years of screening visit
- Persistent or recurrent infections or severe infections requiring hospitalization or treatment with iv antibiotics within 30 days, or oral antibiotics within 14 days prior to enrolment
- Pregnancy or breast-feeding
- Any treatment with hormone replacement therapy or oral contraception.
- Significant renal or hepatic impairment
- Tumour necrosis factor-α (TNF-α) inhibitor other biological agents are not permitted during the trial or within 4 weeks prior to inclusion in the trial. (Infliximab/adalimumab is not permitted 3 months prior to inclusion in the trial; Rituximab is not permitted at least 6 months prior to inclusion in the trial).
- Intramuscular, intra-articular or intravenous injections of corticosteroids are not permitted during the trial or within 4 weeks prior to inclusion in the trial.
- Vaccination with living vaccines is not allowed during the treatment

Patients who met the selection criteria in sections 5.2 and 5.3 will proceed to enrolment.

6 SCHEDULE OF ASSESSMENTS AND PROCEDURES

6.1 Study Procedures

All study data are to be recorded in the source documents and later transcribed onto the case record form (CRF), with the exception of the tender joint count (TJC) and swollen

This document is confidential and must not be communicated to a third party or reproduced or quoted without written consent of the principal investigator.

joint count (SJC), visual analogue scale (VAS) for patient pain, patient global and physician global assessments, health assessment questionnaire (HAQ), which may be documented directly onto the CRF.

The schedule of assessments for the entire study period is presented in Figure 1.

6.2 Informed Consent

At the screening visit the patient will be given a verbal explanation of the study and the procedures involved. Each patient will receive a patient information sheet and will be given enough time to read the information, consider the information given and ask questions. The patient must sign and date a consent form approved by the Regional Ethics Committee before screening procedures are started. A copy of the patient information sheet and consent form will be given to the patient. Further details about how informed consent will be obtained and documented are provided in Section 11.2, Patient Information and Consent.

6.3 Pregnancy Test

In the case of pre-menopausal women, a urine β -hCG test will be performed within 7 days of scheduled visit 1. Patients will only receive study medication in the case of a negative test result.

This document is confidential and must not be communicated to a third party or reproduced or quoted without written consent of the principal investigator.

Figure 1

Visit	0	1	2	3	4
**Day**	**-28**<br>**screen**	**1**<br>**(BL+R)***	**2**	**3**	**4**
Inclusion/Exclusion	X				
Demographics	X				
Signed Informed Consent	X				
Medical History	X				
Vital Signs	X	X	X	X	X
Physical Exam	X				
DAS28 (with 66/68) Assessment	X	X	X	X	X
VAS (pain, global patient/physician)	X	X	X	X	X
HAQ		X			
Pregnancy Test α	X				
Rheumatoid Factor	X	X	X		
Anti-CCP	X	X	X		
CRP/ESR	X	X	X		
Haematology/Chemistry<br>Freeze blood samples	X	X	X		
Urinanalysis (dipstick)	X	X	X		
Cetrotide/Placebo Administration		X	X	X	X
Monitor Adverse Events		X	X	X	X

* BL+R, Baseline and randomisation
α Premenopausal women will only receive study medication in the case of a negative urine β-hCG test result within 7 days prior to scheduled visit 1.

Visit	5	5b[†]	6	7
**Day**	**5**	**5**	**10**	**15**
Inclusion/Exclusion				
Demographics				
Signed Informed Consent				
Medical History				
Vital Signs	X	X	X	X
Physical Exam				X
DAS28 (66/68) Assessment	X	X	X	X
VAS (pain, global patient/physician)	X	X	X	X
HAQ	X	X	X	X
Pregnancy Test (fertile women)				
Rheumatoid Factor		X	X	X
Anti-CCP		X	X	X
CRP/ESR		X	X	X
Haematology/Chemistry<br>Freeze blood samples		X	X	X
Urinanalysis (dipstick)		X	X	X
Cetrotide/Placebo Administration	X			
Monitor Adverse Events	X	X	X	X

6.4 Screening procedures

Patients will be assessed for eligibility by the inclusion and exclusion criteria described in sections 5.2 and 5.3. A screening form will be filled out for eligibility evaluation. Screening assessments are displayed in Figure 1. The screening visit can occur up to 28 days prior to the study medication (except for pregnancy test).

[†] Visit 5b is 12 hours post study medication on day 5 when LH/FSH levels are lowest

This document is confidential and must not be communicated to a third party or reproduced or quoted without written consent of the principal investigator.

6.4.1 DAS28 Assessment

In order to evaluate the eligibility of the patients the treating physician will calculate a DAS28 score at screening. A total of 28 joints will be assessed: shoulders, elbows, wrists, 1st-5th MCP, 1st IP and 2nd-5th PIP joints and knees. Joint tenderness and swollenness is classified as present or absent. The number of tender and/or swollen joints is reported.

DAS28-ESR/CRP can be therefore calculated based on the following formula:

DAS28= 0.56 * sqrt (tender28) + 0.28 * sqrt (swollen28) + 0.014 * VAS global + 0.70 *ln (ESR) or [0.36 *ln (CRP+1) +0.96)]

where sqrt denotes the square root, ESR denotes erythrocyte sedimentation rate in mm/h, CRP denotes C-reactive protein in mg/L, and TJC and SJC are tender joint count and swollen joint count defined as above. Global disease activity measured on a visual analogue scale of 100mm will also be obtained.

6.5 Medical History

A complete medical history (which includes RA related and non-RA related history) will be obtained from each subject during the screening visit. All previous RA related medications will be recorded with maximum duration of treatment, highest doses received, and route of application. Medication (including over-the-counter analgesics and non-steroidal anti-inflammatory drugs [NSAIDs]) use over the 28 days prior to the screening visit, as well as any changes in concomitant medication during the course of the study would also be recorded.

6.6 Baseline Examination/Investigation

Baseline examinations/investigations will be performed according to the schedule of assessments described in Figure 1 on Day 1 prior to drug administration.

This document is confidential and must not be communicated to a third party or reproduced or quoted without written consent of the principal investigator.

6.7 Study Assessments

Complete efficacy and safety assessments will be made on Days 1 (baseline), 2, 5b, 10, and 15.

The sequence of assessments will be as follows:

1. Patient reported assessments: patient's global assessment of disease activity (VAS), pain (VAS), HAQ
2. Other efficacy assessments: joint counts, physicians global assessment of disease activity
3. Safety assessments
4. Laboratory samples for safety and efficacy will be obtained as in Figure 1

6.7.1 Clinical Assessments

The outcome assessor, who is blinded to treatment group, will perform joint assessments at all visits. All joint assessments in the individual patients are planned to be performed by the same assessor. The following assessments will be performed according to the schedule in Figure 1.

- *Patient/Physician assessment of global disease activity:* The patient/physician will indicate their overall ('global') assessment of disease activity on a horizontal VAS (0-100 mm). The scale ranges from 'very well' to 'very poorly'.
- *Patients' assessment of pain (on a Visual Analogue Scale (VAS)):* On a horizontal VAS (0-100 mm), the patients will indicate their current level of pain. The left end (0 mm) signifies no pain and the right end (100 mm) signifies the most pain possible.
- *Patient self-assessed disability (Health Assessment Questionnaire (HAQ)-score):*

- The functional status of the patient will be assessed by means of the disability index of the HAQ. This 20-question instrument assesses the degree of difficulty a person has in accomplishing tasks in 8 functional areas (dressing, arising, eating, walking, hygiene, reaching, gripping and errands/chores). Responses in each functional area are scored from 0 indicating no difficulty, to 3, indicating inability to perform a task in that area. The mean value is reported.
- *Swollen Joint Count/Tender Joint Count:* An assessment of 28 joints for swelling and tenderness will be made. Joints will be assessed and noted as swollen/not swollen and tender/not tender by pressure and joint manipulation on physical examination. Joints which have been replaced, fused, undergone synovectomy at any time point prior to the study will be documented as non evaluable- NE.
- *Duration of morning stiffness:* The duration of morning stiffness at the morning of each visit will be assessed in minutes.

6.7.2 Physical Examination

A general physical examination (including the cardiovascular, respiratory, gastrointestinal and neurological systems) will be performed at the screening visit and final visit. Weight and height will be measured at the screening visit. Findings should be recorded as ‘normal’ or ‘abnormal’ in the source documents and transcribed on the appropriate CRF. Diagnosis of new abnormalities, or worsening abnormalities should be recorded as an adverse event if appropriate.

This document is confidential and must not be communicated to a third party or reproduced or quoted without written consent of the principal investigator.

6.7.3 Vital Signs

Vital sign determinations of sitting blood pressure, heart rate, and body temperature (°C) will be obtained as indicated in the schedule of assessments at screening visit and all subsequent study visits.

6.7.4 Laboratory Assessments

The following laboratory tests will be recorded in the CRF at time points indicated in the Schedule of Assessments, Figure 1. Immediately after collection, blood samples for hormones and other analyses will be processed to serum and stored at -70°C until analysis.

- *Haematology:* Samples for haemoglobin, platelet count and white blood cell count with differential count
- *Clinical Chemistry:* Samples for creatinine and alanine aminotransferase
- *Acute Phase Reactants:* ESR and CRP
- *Urinalysis* Dipstick for protein, blood and sugar will be assessed at screening visit. If protein is detected a quantitative analysis will be performed.
- *Hormones:* Samples for HPG axis hormones, and cortisol
- *Other Analyses:* Cytokine analysis, rheumatoid factor, anti-CCP antibody, bone turnover markers, cardiovascular and endothelial function markers

7 TREATMENT OF SUBJECTS

7.1 Cetrorelix Treatment and Rationale

Studies suggest that a rapid and significant lowering of GnRH (thereby lowering LH and FSH) would give the best clinical response. Cetrorelix is available in a long-acting and short-acting form. For assessing potential therapeutic benefit, the short-acting formulation is

This document is confidential and must not be communicated to a third party or reproduced or quoted without written consent of the principal investigator.

preferred, as it gives a quick substantial lowering of LH and FSH compared to the long-acting formulation. This effect is enhanced by giving repeated injections of cetrorelix. Therefore the intervention arm will receive 5mg/d subcutaneous cetrorelix s.c over the first two days and 3mg/d subcutaneous cetrorelix sc over the following 3 days.

7.1.3 Placebo Treatment

The placebo drug will be 5ml/3 ml (5ml on days 1 and 2; and 3 ml on days 3 through to 5 equalling the same volumes of cetrorelix) subcutaneous sodium chloride s.c. Patients will receive placebo injections with the same schedule as the investigational drug.

7.2 Concomitant treatment

Pateints are allowed to take oral prednisolone (≤ 7.5mg/d) stable dose for at least 4 weeks prior to baseline, NSAIDs if stable for at least 2 weeks and DMARDs if stable for at least 8 weeks. Some patients who do not tolerate/have no effect of MTX therapy or other disease modifying drugs yet have an active disease (DAS28 ≥3.2) may also be allowed to participate. Patients receiving MTX will receive folic acid. This can be as a single dose given weekly or a divided weekly dose. The dosing regime will be recorded in the CRF.

7.3 Rescue Medication

Patients may take stable paracetamol, maximum 1g four times daily at precisely the same time of day. However, patients should not increase or decrease their analgesic doses within 12 hours prior to a visit where clinical efficacy assessments are performed and recorded.

This document is confidential and must not be communicated to a third party or reproduced or quoted without written consent of the principal investigator.

7.4 Administration of the Investigational Drug and Placebo

Administration of placebo or cetrorelix doses will be performed by the research nurse at the study site between 0700 and 0900 hours on Days 1, 2, 3, 4 and 5.

Cetrorelix is a powder and solvent to be made up into a solution for injection. Cetrorelix will only be reconstituted with the solvent provided, using a gentle swirling motion. Vigorous shaking with bubble formation should be avoided. The entire contents of the vial should be withdrawn. The solution should be used immediately after reconstitution. Cetrorelix should be stored below 25° and protected from light. Cetrorelix has a shelf life of 24 months.

All injection sites will be in the lower abdominal wall, around the navel and varied each day. The patient will be kept under medical supervision for 30 minutes after cetrorelix or placebo administration to ensure there is no allergic reaction to the drug. Facilities for treatment of such reactions will be immediately nearby.

7.4 Blinding

This is a double-blind study where both the investigator is unaware of the drug being and the patient is unaware which drug is being received. When the patient enters the treatment room, they will not see the medication packaging. The injection would have been prepared at the nurses' station, away from the patient's room. On entering the room, the patient will only see a colourless liquid in the syringe and will not be able to differentiate whether the colourless, odourless liquid is the test medication or placebo. The nurse will keep a record of what drug has been given which will not be shared with

This document is confidential and must not be communicated to a third party or reproduced or quoted without written consent of the principal investigator.

the investigator until the database lock has occurred after the last patient has had their final visit.

8 SAFETY ISSUES

8.1 Adverse Events

Adverse events (AEs) include the following:

- All suspected adverse drug reactions.

- All reactions from medication overdose, withdrawal, sensitivity or toxicity.

- Apparently unrelated illnesses, including the worsening of a pre-existing illness

- Injury or accidents.

- Abnormalities in physiological testing or physical examination findings that require clinical intervention or further investigation.

-Laboratory abnormalities that require clinical intervention or further investigation unless they are associated with an already reported clinical event.

Pre-existing conditions:

In this trial, a pre-existing condition will not be reported as an AE unless the condition worsens.

Study Disease:

Deterioration in the study disease and signs and symptoms thereof will not be reported as AEs unless the patient discontinues the study due to the sign or symptom or patients meet the specified criteria for stopping the trial (section 9.8).

Adverse Event Reporting Period:

The adverse event-reporting period for this trial begins on inclusion in the trial and ends at the final clinical visit.

Adverse Event Reporting:

This document is confidential and must not be communicated to a third party or reproduced or quoted without written consent of the principal investigator.

Each patient will be questioned about AEs at each clinic visit following initiation of treatment. The investigator is to report all directly observed AEs and all AEs spontaneously reported by the patient. The AE will be noted as either treatment related or other. AEs will be stratified by sex, to map sex specific differences.
AEs spontaneously reported by the patient or observed by the investigator during the trial will be recorded in the CRF.

AEs will be graded according to the Common Terminology Criteria for Adverse Events (CTCAE), version 3. Details of these criteria can be accessed at http://ctep.cancer.gov. AEs not listed by the CTCAE will be graded using the following critera.

Grade 1 (Mild): Discomfort noticed but no disruption of normal daily activity

Grade 2 (Moderate): Discomfort sufficient to reduce or affect normal daily activity

Grade 3 (Severe): Inability to work or perform normal daily activity

Grade 4 (Life-threatening or disabling): Represents an immediate threat to life. Such events should also be reported as serious events.

Relationship of the AE to the treatment should be assessed and noted as follows:

Relationship on AE Form:

- Yes (related)
- No (unrelated)

A ‘yes’ relationship infers that there is a reasonable suspected causal relationship to the trial medication inferring there are facts or arguments to suggest a causal relationship. This does not include methotrexate.

Relationship on AE form:

Related

- Related to cetrorelix

Unrelated

- Pre-existing/underlying disease
- Other treatment (concomitant or previous)
- Protocol related procedure
- Other (accident, new illness)

8.2 Laboratory Abnormalities

An abnormal laboratory result which is clinically significant will be recorded on the AE form in the CRF. Clinically significant results include:

- Those which are accompanied by a clinical symptom, in which case the symptom is recorded as the AE term.
- Those which lead to a change in study medication
- Those which require a change in concomitant therapy

8.3 Handling of Safety Parameters

8.3.1 Serious Adverse Events

The ICH Guideline for Clinical Safety Data Management, Definitions and Standards for Expedited Reporting will be followed. A serious adverse event (SAE) includes any experience which :

- Is fatal
- Is life-threatening
- Requires in-patient hospitalisation
- Results in persistent or significant disability/incapacity

- Is medically significant or requires intervention to prevent one or other of the outcomes listed above

For all adverse events the relationship to the study drug, action taken regarding study drug, and outcome to date will be recorded on the AE form of the CRF.

8.3.2 Serious Adverse Event Reporting

Suspected unexpected serious adverse reactions (SUSARs) will be faxed using the standard CIOMS form (including the EudraCT number of the study) within 24 hours of the study site learning of the event to the Norwegian MA and the drug manufacturer, Asta Medica, Frankfurt, Germany.

8.3.3 Treatment and Follow-up of Adverse Events

AEs should be followed up until they have returned to baseline or stabilised. If the AE has a clear explanation, this should be recorded on the AE in the CRF. Treatment of AEs should follow normal medical standards of care.

8.3.4 Follow-up of Abnormal Laboratory Test Values

If laboratory tests are abnormal and clinically significant as described in 8.2, the tests should be repeated immediately and followed up until they have returned to the normal range and/or an adequate explanation has been found. In the event of an adequate explanation, this should be recorded in the CRF.

8.4 Discontinuation Criteria

A patient should be withdrawn from trial treatment at any time if, in the opinion of the investigator, it is medically necessary, or if it is the wish of the patient. Patients may

This document is confidential and must not be communicated to a third party or reproduced or quoted without written consent of the principal investigator.

withdraw at their own request at any time during the trial period. Should a patient make such a request, the reason should be recorded in the CRF and final visit examinations be performed before addition of new therapy. If a medicine, which is not allowed in the trial, is needed, patients must be withdrawn from the trial. If concomitant therapy needs to be changed in the opinion of the consultant rheumatologist, the patient will be withdrawn from the trial. The consultant rheumatologist, in consultation with the patient, will decide the future course of treatment.

9 STATISTICS

9.1 Sample Size

Assuming a difference of 0.6 unit reduction from baseline in mean DAS28 score on cetrorelix compared with placebo and an underlying standard deviation for the changes from baseline of 1.0 unit (from previous data), we calculated that

$$\mathbf{N = 2(Z\alpha + Z_2\beta)^2\sigma^2 / \delta^2},$$

where $\boldsymbol{\delta = \mu_2 - \mu_1 = 0.6}$

$$\mathbf{N = 2\ (1.96 + 0.842)^2\ 1^2/0.6^2}$$

N= 44 per treatment group

To allow for drop-outs, **49 patients in each group** were considered adequate. The significance is 0.05 (two-tailed) with a power of 0.8.

9.2 Analysis Populations

9.2.1 Safety Population

This population will be used for all summaries of safety data (i.e. adverse events, concomitant medications, concomitant diseases, and laboratory data). The safety

This document is confidential and must not be communicated to a third party or reproduced or quoted without written consent of the principal investigator.

population will include all patients who are randomised and received any injections of study drug.

9.2.2 Intention to Treat (ITT) Population

This population will be used for all efficacy analyses. All randomised patients who have received any injections of study drug will be included in this population.

9.2.3 Per Protocol Population

The per-protocol population will be used only for the analysis of the primary endpoint as a sensitivity check to the ITT analysis, and in order to evaluate the influence of major protocol violations and protocol deviators. Patients may be excluded if they significantly violate the inclusion/exclusion criteria or deviate from the study plan. Patients who receive incorrect therapy from that intended will be excluded from the per-protocol population.

9.3 Statistical and Analysis Methods

The study period is 15 days and the statistical analyses will be performed on the data collected up to this point. Once all patients have completed the trial, a database lock will occur. Treatment assignments will be primarily unblinded for the study statistician for the purpose of data analysis.

All analyses, summaries and listings will be performed using statistical software, including Stata 11 (StatCorp LP, College Station, TX) and StatXact 9 (Cytel Inc., Cambridge, MA). For both intervention arms, means and their corresponding standard deviations (SD) will be computed to describe the distributions of demographic and

baseline variables. Means and SDs will also be used to describe distributions of corresponding changes from baseline. Distributions for categorical variables will be described for each group with frequencies and/or percentages. Medians and quartiles will describe percent changes. Two-sided tests and confidence intervals will be used at all end points. Because of the unpredictability of some of the problems that may arise with regards to data collection, detailed consideration of the handling of unforeseen irregularities (e.g. outliers) will be deferred until blind review of the data at the end of the trial.

9.3.1 Assessment of Treatment Response

Based on VAS, HAQ, ESR, CRP, and Joint Count Scores, a number of disease activity scores (DAS28, EULAR response, ACR 20/50/70/90 response) will be calculated for each visit and used for the assessments of treatment response.

DAS28CRP:

See earlier for calculation of this

ACR definition of improvement:

‘ACR20’, ‘ACR50’, ‘ACR70’, and 'ACR90’ are defined as 20%, 50%, 70%, and 90% improvement from baseline, respectively, in:

- Tender Joint Count (TJC) and
- Swollen Joint Count (SJC) and

and in 3 of the following 5 assessments:

- Patient Pain Assessment (VAS Pain)
- Patient Global Assessment (VAS Global)

This document is confidential and must not be communicated to a third party or reproduced or quoted without written consent of the principal investigator.

- Physician Global Assessment (VAS Physician)
- Patient Self-Assessed Disability (HAQ)
- Acute Phase Reactant (CRP or ESR)

EULAR response criteria:

The EULAR response criteria are based on attained level and change in DAS, classifying patients as good, moderate or non responders, as follows:

DAS28 at endpoint	Improvement in DAS28 from baseline		
	>1.2	>0.6 and ≤ 1.2	≤0.6
≤ 3.2	Good	Moderate	None
> 3.2 and ≤ 5.1	Moderate	Moderate	None
>5.1	Moderate	None	None

The presence of ‘good’, ‘moderate’ or ‘no response’ will be determined for each visit.

Hypothesis testing

The cetrotide arm will be considered superior to placebo if there is enough statistical evidence to reject the following null hypothesis:

Ho: $\mu1= \mu2$ (i.e. adjusted change in DAS28CRP scores in the cetrotide and placebo arms are equal),

and accept the alternative hypothesis:

Ho: $\mu1\neq \mu2$ (i.e. the adjusted change in DAS28CRP score in the cetrotide arm is superior to the change in DAS28 score in the placebo arm).

If the test result is statistically significant at α=0.05 level, we will conclude that the cetrotide arm demonstrated a superior change in DAS28CRP score at day 5b when compared to placebo.

9.3.2 Primary Endpoint and Analysis

Cetrorelix has a rapid onset and offset effect; therefore we have chosen an early primary endpoint. The primary endpoint is the change in disease activity score (DAS28CRP) on Day 5b (12 hours after the last injection [time point when LH and FSH levels are expected to be lowest]).

The primary variable will be analysed by ANCOVA, linear regression with last measurement (in this case day 5b) as the dependent variable and treatment and baseline measurement as independent variables (covariates).

9.3.3 Secondary Endpoints and Analysis

DAS28 refers to both DAS28ESR and DAS28CRP

Secondary Endpoints:

1. Change in DAS28 levels from baseline to days 2, 10, and 15 will be analyzed using ANCOVA (see section 9.3.2).
2. The change in cytokine levels, cardiovascular markers, endothelial function markers and bone markers from baseline to day 2, 5, 10 and 15 will be analyzed using ANCOVA in a similar manner as the DAS28 levels (see section 9.3.2).
3. The change in ACR core set measures from baseline to day 2, 5, 10 and 15 will be analyzed using ANCOVA in a similar manner as the DAS28 levels (see section 9.3.2).

This document is confidential and must not be communicated to a third party or reproduced or quoted without written consent of the principal investigator.

4. The proportion of patients with ACR20/50/70/(90) response on day 2, 5, 10 and 15 will be analyzed using the Pearson's χ^2 test (or Suissa Shuster exact unconditional test[44])
5. The proportion of patients with EULAR response rate ('Good', 'Moderate' or 'None') from baseline to day 2, 5, 10 and 15.The Pearson's χ^2 test for trend for comparisons of proportions among ordered groups (or Suissa Shuster exact unconditional test) will be used to assess the difference in proportions between groups.
6. DAS28CRP low disease activity on day 2, 5, 10 and 15 (defined as DAS28 $\leq$3.2) will be analyzed using the Pearson's χ^2 test (or Suissa Shuster exact unconditional test)
7. DAS28CRP remission on day 2, 5, 10 and 15 (defined as DAS28 $<$2.6) will be analyzed using the Pearson's χ^2 test (or Suissa Shuster exact unconditional test)
8. The correlation between relative changes in hormones, disease activity and biomarkers will be assessed using the Spearman rank correlation.
9. The change in HAQ scores from baseline to day 5, 10 and 15 will be analyzed using ANCOVA in a similar manner as the DAS28 levels (see section 9.3.2).
10. The change in anti-CCP levels from baseline to day 2, 5, 10 and 15 will be analyzed using ANCOVA in a similar manner as the DAS28 levels (see section 9.3.2).
11. Number of patients with AEs (and SAEs) up to day 15 in each group will be analyzed using the Pearson's χ^2 test (or Suissa Shuster exact unconditional test)

The variables described in the secondary endpoints are highly correlated and therefore no adjustment has been made for multiple testing.

This document is confidential and must not be communicated to a third party or reproduced or quoted without written consent of the principal investigator.

Appropriateness of the statistical methodology

The appropriateness of the statistical model for each of the variables outlines in the sections, ‘Primary Endpoint and Analysis’ and ‘Secondary Endpoint and Analysis’ be assessed prior to conducting analyses. This would include, but is not limited to, checking for outliers, checking the histogram for normal distribution, checking the homogeneity of variance and checking expected cell frequencies. If necessary, transformation of severely skewed variables and/or use of nonparametric alternative analyses will be performed.

9.4 Missing Data

Missing data will be replaced using the last observation carried forward (LOCF) method. The last observed value will be used to impute all the missing values for that person. In the event of substantial missing data (>10% of the expected data set), multiple imputation will be used as an additional method for handling missing values. The purpose of carrying out multiple imputation would be to provide a sensitivity analysis to the LOCF method. If there is a significant difference between these methods, the multilple imputation approach is considered to be more reliable for large amounts of missing data. A previous study supported the use of modern imputation techniques as opposed to simpler techniques such as the LOCF method or case deletion method.[45] Multiple imputation has also been recommended for use in RA trials for a variety of outcome measures including measures of disease activity. [46]

This document is confidential and must not be communicated to a third party or reproduced or quoted without written consent of the principal investigator.

9.5 Safety Data Analyses

All AEs will be coded and tabulated by body system and the preferred term for individual events within each body system and will be presented in descending frequency. AEs will also be tabulated by severity and relationship to cetrorelix. SAEs will be summarised separately.

Associated laboratory tests such as haematology, liver function tests, kidney function tests will be grouped and presented together. For each laboratory test, all individual patient values will be listed and values outside the standard reference range will be highlighted. Marked abnormalities will be tabulated for each laboratory test by treatment group.

The change from baseline for each of the vital sign variables for each individual patient will be listed and summarised using descriptive statistics.

9.6 Interim Analysis

No interim analyses have been planned.

9.7 Replacement Policy

No patient who discontinues the study for whatever reason will be replaced.

9.8 Criteria for termination of the trial

The trial will be terminated if there are unacceptable side effects of the intervention such as a serious drug related AE or if there is a significant worsening of the RA disease (increase in DAS28 of more than 3.2 points) by day 3 in a large proportion of patients.

This document is confidential and must not be communicated to a third party or reproduced or quoted without written consent of the principal investigator.

The investigator (sponsor) reserves the right to terminate the study at any time. In terminating the study, adequate consideration will be given to the protection of the patients' interests.

10 MONITORING AND QUALITY ASSURANCE

Data from this study will be recorded via CRFs which will then be transferred onto an electronic database. Source document review will be performed against the entries on the CRF and a quality assurance check (by an independent data-monitoring committee, Ullevål University Hospital Data Monitoring Committee) will be performed to ensure that the investigator is complying with the protocol and regulations as outlined in a monitoring plan. The monitor will have access to laboratory results, and other patient records to verify the entries on the CRF.

Routine haematology, serum chemistry and urine analysis will be conducted locally and the results will be entered into the CRF when appropriate. Two central laboratories will conduct the hormone and immunological analyses. The data from these blinded analyses will be electronically transferred from the central laboratories to the study database.

For classification purposes, preferred terms will be assigned for the original terms entered on the CRF using MedDRA (Medical Dictionary for Regulatory Activities).

The Norwegian Medicines Agency (MA), national health authority, and local health authority are permitted to inspect facilities and records relevant to this study.

This document is confidential and must not be communicated to a third party or reproduced or quoted without written consent of the principal investigator.

11 ETHICS

11.1 Ethical conduct of trial

Good Clinical Practice (GCP) requires that the clinical protocol, any protocol amendments, the Investigator's Brochure, the informed consent and all other forms of subject information related to the study and any other necessary documents to be reviewed by an Ethical Committee (EC) and MA. Any amendments to the protocol will require EC/MA approvals prior to implementation of any changes made to the study design.

During the conduct of the study, the investigator will promptly provide written reports to the EC/MA of any changes that affect the conduct of the study and/or increase the risk to subjects.

11.2 Patient Information and Consent

It is the responsibility of the investigator or representative to give each subject prior to inclusion in the trial, full and adequate verbal and written information regarding the objective and procedures of the trial and the possible risks involved, and answer all questions regarding this study. The meeting will take place at an undisturbed location. The subjects will be informed about their right to withdraw from the trial at any time. Prior to any screening procedures being performed on the subject, the informed consent statement will be reviewed and signed and dated by the subject and the person who administered the informed consent. A copy of the informed consent form will be given to the subject and the original will be placed in the subject's medical record.

This document is confidential and must not be communicated to a third party or reproduced or quoted without written consent of the principal investigator.

11.3 Withholding treatment

Patients with RA will not be withheld treatment because the common disease modifying anti-rheumatic drug (DMARD) treatment for RA patients is allowed in the trial. Patients who have previously failed on biologic therapy may also take part in the trial providing an adequate wash-out period has elapsed by time of screening. The short duration of the trial also allows patients with high disease activity to participate, without postponing the initiation of a new drug for a long period of time.

12 DATA HANDLING AND RECORD KEEPING

The investigator will keep records and original signed consent forms. To comply with international regulations, the investigator will retain the records for 15 years.

Documents will be classified into two main categories: 1. Investigator’s Study File, and 2. Subject Clinical Source Documents. The Investigator’s Study File will contain the protocol and its amendments, EC approval with correspondence, MA approval with correspondence, sample informed consent, and other relevant documents. The Subject’s Clinical Source Documents will include patient hospital records, physician’s/nurses notes, original laboratory reports, signed informed consent, subject screening and enrolment forms.

Below is an example of the table to be used to log the medication given to patients. The research nurse is responsible for the completion of this table.

This document is confidential and must not be communicated to a third party or reproduced or quoted without written consent of the principal investigator.

Medication Log

Patient ID No./Initials	Patient Medication Number	Patient Randomisation Number	Name of Drug	Dose	Batch No./ Expiry Date	Amount

13 PUBLICATION POLICY

In all publications concerning this study, financial supporters will be acknowledged.

This document is confidential and must not be communicated to a third party or reproduced or quoted without written consent of the principal investigator.

14 REFERENCES

[1] Alamanosa Y, Drosos AA. Epidemiology of adult rheumatoid arthritis. *Autoimmun Rev* 2005; **4**:130–6.
[2] KW Drossaers-Bakker, M de Buck, D van Zeben, AH Zwinderman, FC Breedwald and JM Hazes. Long-term course and outcome of functional capacity in rheumatoid arthritis: the effect of disease activity and radiologic damage over time. *Arthritis Rheum* 1999; **42**:1854–60.
[3] T Pincus, T Sokka and F Wolfe. Premature mortality in patients with rheumatoid arthritis: evolving concepts. *Arthritis Rheum* 2001; **44**:1234–6.
[4] Wilder RL. Neuroendocrine-immune system interactions and autoimmunity. *Annu Rev Immunol* 1995; **13**: 307–38.
[5] Blalock JE. A molecular basis for bidirectional communication between the immune and neuroendocrine systems. *Physiol Rev* 1989; **69**:1–32.
[6] Goetzl EJ, Sreedharan SP. Mediators of communication and adaptation in the neuroendocrine and immune system. *FASEB J* 1992; **6**: 2646–52.
[7] Reichlin S. Neuroendrocrine-immune interactions. *N Eng J Med* 1993; **329**:1246–53.
[8] Rudge SR, Kowanko IC, Drury PL. Menstrual cyclicity of finger joint size and grip strength in patients with rheumatoid arthritis. *Ann Rheum Dis* 1983; **42**: 425–30.
[9] Østensen M, Aune B, Husby G. Effect of pregnancy and hormonal changes on the activity of rheumatoid arthritis. *Scand J Rheumatol* 1983; **12:** 69-72.
[10] Silman A, Kay A, Brennan P. Timing of pregnancy in relation to the onset of rheumatoid arthritis. *Arthritis Rheum* 1992; **35**:152–5.
[11] Goemaere S, Ackerman C, Goethals K, d'Keyser F, D van Straeten, Verbruggen G, Mielants H, Veys EM. Onset of symptoms of rheumatoid arthritis in relation to age, sex and menopausal transition. *J Rheumatol* 1990; **17**:1620–2.
[12] Chen H-F, Jeung E-B, Stephenson M, Leung PCK. Human peripheral blood mononuclear cells express gonadotropin-releasing hormone (GnRH), GnRH receptor, and interleukin-2 receptor γ-chain messenger ribonucleic acids that are regulated by GnRH in vitro. *J Clin Endocrinol Metab* 1999; **84**:743–50.
[13] Silveira LF, Stewart PM, Thomas M, Clark DA, Bouloux PM, MacColl GS. Novel homozygous splice acceptor site GnRH receptor (GnRHR) mutation: Human GnRHR "Knockout". *J Clin Endocrinol Metab* 2002; **87**: 2973–7.
[14] Azad N, LaPaglia N, Jurgens KA, Kirsteins L, Emanuele NV, Kelley MR, Lawrence AM, Mohagheghpour N. Immunoactivation enhances the concentration of luteinizing hormone-releasing hormone peptide and its gene expression in human peripheral T-lyphocytes. *Endocrinology* 1993; **133**: 215–23.
[15] Azad N, LaPaglia N, Kirsteins L, Uddin S, Steiner J, Williams DW, Lawrence AM, Emanuele MV. Jurkat cell proliferative activity is increased by luteinizing hormone-releasing hormone. *J Endocrinol* 1997; **153**: 241–9.
[16] Marchetti B, Guarcello V, Morale MC, Bartoloni G, Raiti F, Palumbo G, Farinella Z, Cordaro S, Scapagnini U. Luteinizing hormone-releasing hormone (LHRH) agonist restoration of age-associated decline of thymus weight, thymic LHRH receptors, and thymocyte proliferative capacity. *Endocrinology* 1989; **125**:1037-45.
[17] Morale MC, Batticane N, Bartoloni G, Guarcello V, Farinella Z, Galasso MG, Marchetti B. Blockade of central and peripheral luteinizing hormone-releasing hormone (LHRH) receptors in neonatal rats with a potent LHRH-antagnoist inhibits the morphofunctional development of the thymus and maturation of the cell-mediated and humoral immune responses. *Endocrinology* 1991; **128**: 1073-85.
[18] Jacobson JD, Nisula BC, Steinberg AD. Modulation of the expression of murine lupus by gonadotropin-releasing hormone analogues. *Endocrinology* 1994; **134**: 2516-23.
[19] Sabharwal P, Varma S, Malarkey WB. Human thymocytes secrete luteinizing hormone: An autocrine regulator of T cell proliferation. *Biochem Biophys Res Comm* 1992; **187**:1187–92.
[20] Iqbal J, Sun Li, Kumar TR, Blair HC, Zaidi M. Follicle-stimulating hormone stimulates TNF production from immune cells to enhance osteoblast and osteoclast formation. *Proc Natl Acad Sci USA* 2006; **103**:14925–30.
[21] Kåss A, Lea T, Torjesen P, Gulseth HC, Førre Ø. The association of luteinizing hormone and follicle-stimulating hormone with cytokines and markers of disease activity in rheumatoid arthritis: a case-control study. *Scand J Rheumatol* 2010; **39**: 109–17.
[22] Committee for Proprietary Medicinal Products European Public Assessment Report (EPAR) Cetrotide. The European Agency for the Evaluation of Medicinal Products ,13 April 1999 CPMP/2979/98.
[23] Gonzalez-Barcena D, Vadillo Buenfil M, Garcia Procel E, Guerra-Arguero L, Cardenas Comejo I, Comaru-Schally AM, et al. Inhibition of luteinizing hormone, follicle-stimulating hormone and sex-steroid levels in men and women with a potent antagonist analogue of luteinizing hormone-releasing hormone, cetrorelix (SB-75). *Eur J Endocrinol* 1994; **131**: 286–92.

This document is confidential and must not be communicated to a third party or reproduced or quoted without written consent of the principal investigator.

[24] Duijkers IJ, Klipping C, Willemsen WN, Krone D, Schneider F, Niebch G, et al. Single and multiple dose pharmokinetics and pharmacodynamics of the gonadotropin-releasing hormone antagonist cetrorelix in healthy female volunteers. *Hum Reprod* 1998; **13**: 2392–8.
[25] Sommer L, Zanger K, Dyong T, Dorn C, Luckhaus J, Diedrich K et al. Seven day administration of the gonadotropin-releasing hormone antagonist cetrorelix in normal cycling women. *Eur J Endocrinol* 1994; **131**: 280–5.
[26] Leroy I, d'Acremont MF, Brailly-Tabrd S, Frydman R, de Mouzon J, Bouchard P. A single injection of gonadotropin-releasing hormone (GnRH) antagonist (cetrorelix) postpones the luteinizing hormone (LH) surge: further evidence for the role of GnRH during the LH surge. *Fertil Steril* 1994 ; **62**: 461–7.
[27] Behre HM, Klein B, Steinmeyer E, McGregor GP, Viogt K, Nieschlag E. Effective suppression of luteinizing hormone and testosterone by single doses of the new gonadotropin-releasing hormone antagonist Cetrorelix (SB-75) in normal men. *J Clin Endocrinol Metab* 1992; **75**: 393–8.
[28] Gonzalez-Barcena D, Vadillo Buenfil M, Garcia Procel E, Guerra-Arguero L, Cardenas Comejo I, Comaru-Schally AM et al. Inhibition of luteinizing hormone, follicle-stimulating hormone and sex-steroid levels in men and women with a potent antagonist analogue of luteinizing hormone-releasing hormone, cetrorelix (SB-75). *Eur J Endocrinol* 1994; **131**: 286–92.
[29] Comaru-Schally A M, Brannan W, Schally AV, Colcolough M, Monga M. Efficacy and safety of luteinizing hormone-releasing hormone antagonist cetrorelix in the treatment of symptomatic benign prostatic hyperplasia. *J Clin Endocrinol Metab* 1998; **83**: 3826–31.
[30] Stangelberger A, Schally AV, Zarandi M, Heinrich E, Groot K, Havt A, Kanashiro CA et al. The combination of antagonists of LHRH with antagonists of GHRH improves inhibition of androgen sensitive MDA-PCa-2b and LuCaP-35 prostate cancers. *Prostate* 2007; **67**: 1339–53.
[31] Gonzalez-Barcena D, Vadillo-Buenfil M, Gomez-Orta F, Fuentes Garcia M, Cardenas-Cornejo I, Graef-Sanchez A, Comaru-Schally AM et al. Responses to the antagonistic analog of LH-RH (SB-75, Cetrorelix) in patients with benign prostatic cancer. *Prostate* 1994; **24**: 84–92.
[32] Comaru-Schally A M, Brannan W, Schally AV, Colcolough M, Monga M. Efficacy and safety of luteinizing hormone-releasing hormone antagonist cetrorelix in the treatment of symptomatic benign prostatic hyperplasia. *J Clin Endocrinol Metab* 1998; **83**:3826–31.
[33] Stangelberger A, Schally AV, Zarandi M, Heinrich E, Groot K, Havt A, Kanashiro CA et al. The combination of antagonists of LHRH with antagonists of GHRH improves inhibition of androgen sensitive MDA-PCa-2b and LuCaP-35 prostate cancers. *Prostate* 2007; **67**: 1339–53.
[34] Gonzalez-Barcena D, Vadillo-Buenfil M, Gomez-Orta F, Fuentes Garcia M, Cardenas-Cornejo I, Graef-Sanchez A, Comaru-Schally AM et al. Responses to the antagonistic analog of LH-RH (SB-75, Cetrorelix) in patients with benign prostatic cancer. *Prostate* 1994; **24**: 84–92.
[35] Behre HM, Böckers A, Schlingheider A, Nieschlag E. Sustained suppression of serum LH, FSH and testosterone and increase of high-density lipoprotein cholesterol by daily injections of the GnRH antagonist cetrorelix over 8 days in normal men. *Clin Endocrinol (Oxf)* 1994;**40**:241–8.
[36] Committee for Proprietary Medicinal Products European Public Assessment Report (EPAR) Cetrotide. The European Agency for the Evaluation of Medicinal Products, 13 April 1999 CPMP/2979/98.
[37] Gonzalez-Barcena D, Vadillo Buenfil M, Garcia Procel E, Guerra-Arguero L, Cardenas Comejo I, Comaru-Schally AM et al. Inhibition of luteinizing hormone, follicle-stimulating hormone and sex-steroid levels in men and women with a potent antagonist analogue of luteinizing hormone-releasing hormone, cetrorelix (SB-75). *Eur J Endocrinol* 1994; **131**: 286–92.
[38] Duijkers IJ, Klipping C, Willemsen WN, Krone D, Schneider F, Niebch G et al. Single and multiple dose pharmokinetics and pharmacodynamics of the gonadotropin-releasing hormone antagonist cetrorelix in healthy female volunteers. *Hum Reprod* 1998;**13**:2392–8.
[39] Sommer L, Zanger K, Dyong T, Dorn C, Luckhaus J, Diedrich K et al. Seven day administration of the gonadotropin-releasing hormone antagonist cetrorelix in normal cycling women. *Eur J Endocrinol* 1994;**131**: 280–5.
[40] Leroy I, d'Acremont MF, Brailly-Tabrd S, Frydman R, de Mouzon J, Bouchard P. A single injection of gonadotropin-releasing hormone (GnRH) antagonist (cetrorelix) postpones the luteinizing hormone (LH) surge: further evidence for the role of GnRH during the LH surge. *Fertil Steril* 1994 ; **62**: 461–7.
[41] Leroy I, d'Acremont MF, Brailly-Tabrd S, Frydman R, de Mouzon J, Bouchard P. A single injection of gonadotropin-releasing hormone (GnRH) antagonist (cetrorelix) postpones the luteinizing hormone (LH) surge: further evidence for the role of GnRH during the LH surge. *Fertil Steril* 1994 ; **62**: 461–7.
[42] Dubourdieu S, Charbonnel B, D'Acremont M-F, Carreau S, Spitz, IM, Bouchard P. Effect of administration of a gonadotropin-releasing hormone (GnRH) antagonist (Nal-GLu) during the periovulatory period: The luteinizing hormone surge requires secretion of GnRH. *J Clin Endocrinol Metab* 1994; **78**: 343–7.

This document is confidential and must not be communicated to a third party or reproduced or quoted without written consent of the principal investigator.

[43] Küpker W, Felberbaum RE, Krapp M, Schill T, Malik E, Diedrich K. Use of GnRH antagonists in the treatment of endometriosis. *Reprod Biomed Online* 2002; **5**: 12–6.
[44] Lydersen S, Fagerland MW, Laake P. Recommended tests for association in 2*2 tables. *Stat Med* 2009; **28**: 1159–75.
[45] Baron G, Ravaud P, Samson A, Giraudeau B. Missing data in randomised controlled trials of rheumatoid arthritis with radiographic outcomes: a simulation study. *Arthritis Rheum* 2008; **59**: 25–31.
[46] Boers M. Missing data in trials: Do we have to keep carrying the last observation carried forward? *Arthritis Rheum* 2008; **59**: 2–3.
